# Access to New Cytotoxic Triterpene and Steroidal Acid-TEMPO Conjugates by Ugi Multicomponent-Reactions †

**DOI:** 10.3390/ijms22137125

**Published:** 2021-07-01

**Authors:** Haider N. Sultani, Ibrahim Morgan, Hidayat Hussain, Andreas H. Roos, Haleh H. Haeri, Goran N. Kaluđerović, Dariush Hinderberger, Bernhard Westermann

**Affiliations:** 1 Department of Bioorganic Chemistry, Leibniz-Institute of Plant Biochemistry, Weinberg 3, 06120 Halle, Germany; haidersoltani@yahoo.com (H.N.S.); Ibrahim.morgan@ipb-halle.de (I.M.); Hidayat.Hussain@ipb-halle.de (H.H.); goran.kaluderovic@hs-merseburg.de (G.N.K.); 2 Physical Chemistry—Complex Self-Organizing Systems, Institute of Chemistry, Martin Luther University Halle-Wittenberg, von-Danckelmann-Platz 4, 06120 Halle, Germany; Andreas.roos@chemie.uni-halle.de (A.H.R.); haleh.hashemi-haeri@chemie.uni-halle.de (H.H.H.); dariush.hinderberger@chemie.uni-halle.de (D.H.); 3 Department of Engineering and Natural Sciences, University of Applied Sciences Merseburg, Eberhard-Leibnitz-Strasse 2, 06217 Merseburg, Germany; 4 Organic Chemistry, Institute of Chemistry, Martin-Luther University Halle-Wittenberg, Kurt-Mothes-Strasse 2, 06120 Halle, Germany

**Keywords:** multi-component reaction, fusidic acid, TEMPO-conjugate, electron paramagnetic resonance (EPR) spectroscopy, caspase-3

## Abstract

Multicomponent reactions, especially the Ugi-four component reaction (U-4CR), provide powerful protocols to efficiently access compounds having potent biological and pharmacological effects. Thus, a diverse library of betulinic acid (BA), fusidic acid (FA), cholic acid (CA) conjugates with TEMPO (nitroxide) have been prepared using this approach, which also makes them applicable in electron paramagnetic resonance (EPR) spectroscopy. Moreover, convertible amide modified spin-labelled fusidic acid derivatives were selected for post-Ugi modification utilizing a wide range of reaction conditions which kept the paramagnetic center intact. The nitroxide labelled betulinic acid analogue **6** possesses cytotoxic effects towards two investigated cell lines: prostate cancer PC3 (IC_50_ 7.4 ± 0.7 μM) and colon cancer HT29 (IC_50_ 9.0 ± 0.4 μM). Notably, spin-labelled fusidic acid derivative **8** acts strongly against these two cancer cell lines (PC3: IC_50_ 6.0 ± 1.1 μM; HT29: IC_50_ 7.4 ± 0.6 μM). Additionally, another fusidic acid analogue **9** was also found to be active towards HT29 with IC_50_ 7.0 ± 0.3 μM (CV). Studies on the mode of action revealed that compound **8** increased the level of caspase-3 significantly which clearly indicates induction of apoptosis by activation of the caspase pathway. Furthermore, the exclusive mitochondria targeting of compound **18** was successfully achieved, since mitochondria are the major source of ROS generation.

## 1. Introduction

Reactive oxygen species (ROS) are involved in numerous processes, which mediate physiological and pathophysiological signal transductions. Upon unregulated increased ROS production, redox imbalances occur, which cause atherosclerosis, cardiovascular diseases, hypertension, diabetes mellitus, neurodegenerative and immune-inflammatory diseases. In addition, the impact of oxidants and antioxidants in tumor cell proliferation is observed frequently. On the molecular level, ROS causes oxidative stress, which is responsible for damaging cell structures by acting on lipids, membranes, proteins, and DNA. This behavior of ROS in cancer cells, in particular, offers a basis for the prevention of tumor progression and metastasis by ROS scavengers [1,2]. Therefore, antioxidant therapies are sought to selectively inhibit the growth of tumor cells to induce cellular differentiation and to alter the intracellular redox state [3]. Besides this, antioxidative food supplements enhance the positive outcome of conventional cancer treatments. In the context of cancer therapy, nitroxide-modified natural products have been shown to be useful due to their ability to remove superoxide anions, trap carbon-centered radicals, or to terminate chain reactions [4]. Furthermore, these derivatizations have been applied in bioreductive drugs to initiate additional cytotoxic events, which make them useful as antitumor drugs [5].

**Figure 1:**
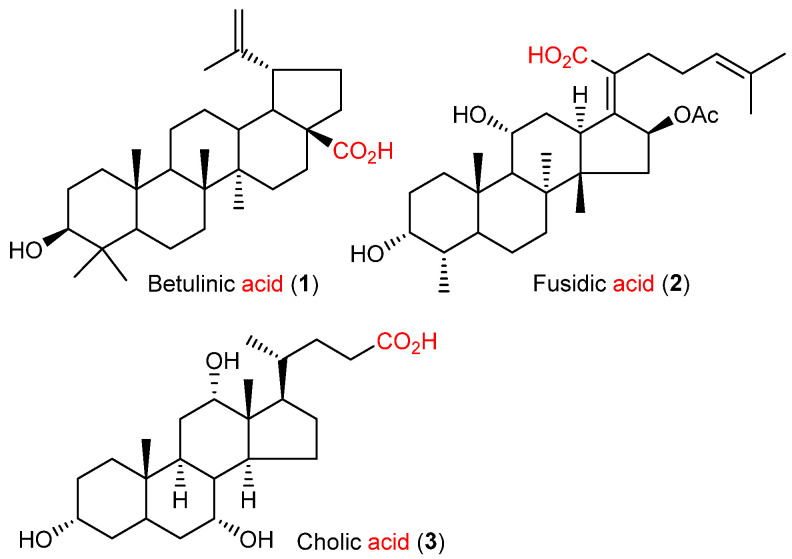
Structures of Betulinic acid (**1**), Fusidic acid (**2**), and Cholic acid (**3**).

Betulinic acid (BA, **1**, Figure 1) and its congeners are well known for their abilities to act as natural cytotoxic products [6,7,8,9,10]. These triterpenes have been modified quite extensively providing products with enhanced biological activities [11,12,13,14,15]. Modifications of these lupane triterpenoids with nitroxyl radicals have been shown to produce a positive outcome on the cytotoxic activity on several cell lines (e.g., CEM13, U937, MT4) [16,17]. Fusidic acid (FA, **2**) is a triterpene acid that belongs to the family of tetracyclic fusidane nor-triterpenes and has been clinically employed as an antibiotic for staphylococcal infections [18]. Additionally, it has been reported that fusidic acid sodium salt, an approved bacteriostatic antibiotic, showed significant cytotoxic effects (in vitro and in vivo) towards various colon cancer cells alone or coupled with 5-fluorouracil [19,20]. Moreover, various studies have been reported that cholic acid (CA, **3**), which is steroidal acid, can be used for the prevention and treatment of colon cancer [21,22].

**Scheme 1:**
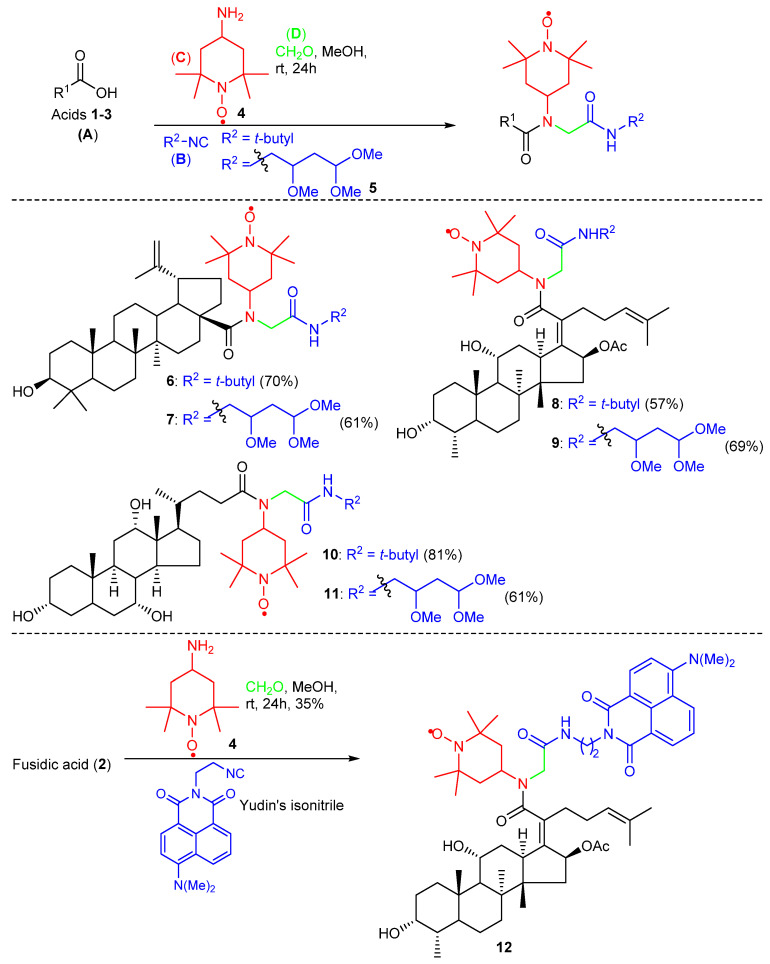
Synthesis of terpenoic acid-TEMPO adducts **6**–**12**.

Based on these promising cytotoxic effects of the natural products **1**–**3**, we prepared a library of BA (**1**), FA (**2**), CA (**3**) conjugates with TEMPO (nitroxide) by utilizing an Ugi multicomponent reaction approach (U-4CR) with the aim of enhancing the cytotoxic potentials of our conjugates. Although only a few reports have been published about the U-4CR modifications on BA (**1**) as anti-inflammatory agents [23,24], none of them investigated the fusion to nitroxide. In a previous communication, we demonstrated that the U-4CR strategy is very well suited to achieve spin-labelled products [25]. In the present study, we use an amino spin-label viz.: 4-amino-2,2,6,6-tetramethylpiperidine-1-oxyl (**4**, 4-NH_2_-TEMPO) as a U-4CR counterpart (Scheme 1) allowing for the preparation of the natural acid-TEMPO adducts. The spin-labelled FA derivative **8** acts strongly against two investigated cancer cell lines of prostate cancer (PC3) and colon cancer (HT29) and induces apoptosis by a caspase-dependent mechanism.

## 2. Results and Discussion

### 2.1. Chemistry

The general synthetic pathway for the preparation of natural acids **1**–**3**-based Ugi products **6**–**12** is outlined in Scheme 1. Natural acid-TEMPO adducts **6**–**11** were synthesized in a single step operation by utilizing the Ugi four-component reaction. These compounds were prepared in moderate to good yields (57–81%) by the reaction of BA (**1**), FA (**2**), or CA (**3**) as the acid component (A), convertible IPB isonitrile **5** [26], or *t*-butyl isocyanide as isonitrile component (B), 4-NH_2_-TEMPO as amine (C), and paraformaldehyde (D) in the presence of MeOH. Encouraged by our previous results [25] that spin-label TEMPO is not affected under the reaction conditions of the U-4CR, we plan to couple nitroxide comprising amine viz.: 4-NH_2_-TEMPO (**4**; as amine component) to enhance the cytotoxicity of natural acids **1**–**3**. Moreover, BA (**1**), FA (**2**), and CA (**3**) have a tertiary carboxylic acid, vinyl carboxylic acid, and secondary carboxylic acid groups respectively. We found that the alteration of these acids did not play any significant role in the product yields. To introduce further chemical diversity via the Ugi synthetic procedure, we additionally prepared fusidic acid-based Ugi product **12** by utilizing Yudin’s fluorescent isocyanide [27].

**Scheme 2:**
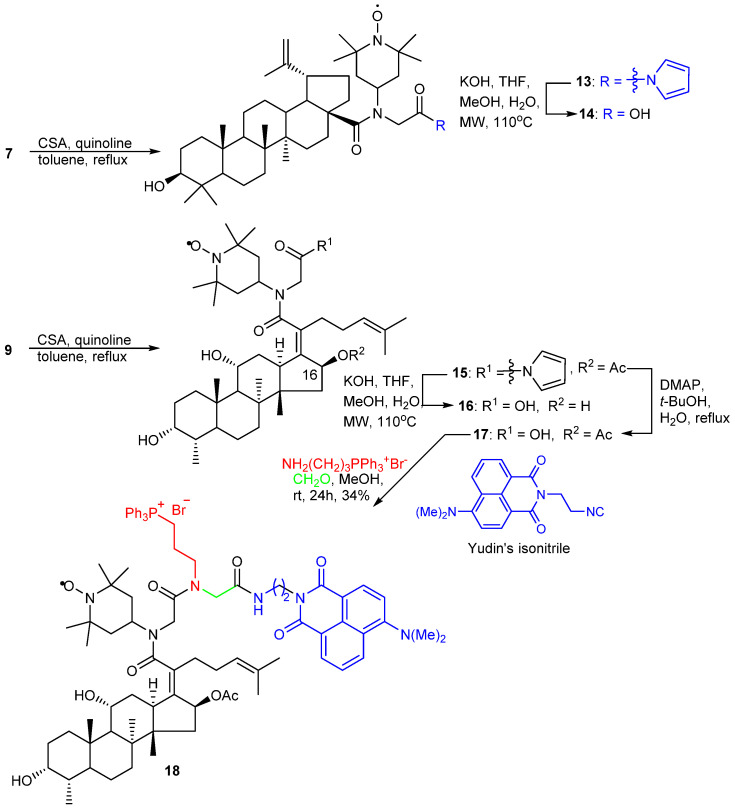
Synthesis of compounds **13**–**18**.

In order to expand the diversity of the Ugi products synthesized, the advantage was taken of the isonitrile functionality as illustrated for compounds **7** and **9** (Scheme 2). As demonstrated earlier [26], the secondary amide can be transformed upon acidic treatment to acyl pyrroles, which can easily be cleaved by nucleophiles. Thus, in the presence of camphor sulfonic acid (CSA) both Ugi-products **7** and **9** were transformed to the corresponding acyl pyrroles **13** and **15**, which upon treatment with KOH were converted into the corresponding carboxylates **14** and **16**. However, no selectivity could be obtained for the acetyl moiety in the fusidic acid derivative **15** since the ester moiety (C-16 acetyl group) was cleaved as well, as expected under these conditions. To achieve selectivity in the displacement of the acyl pyrrole, an alternative procedure (DMAP, H_2_O/*t*-BuOH) was successful and furnished the C-16 acetyl fusidic acid analog **17**. For biological evaluation, we envisioned the preparation of a conjugate with a triphenylphosphine moiety, since this moiety is known to selectively bind to mitochondria membranes. Again, the U-4CR proved to be the synthetic protocol of choice, since in a single step not only the triphenylphosphine moiety but a dye (Yudin’s dye/Yudin’s isonitrile) can be assembled to form the product in the same synthetic process yielding the fusidic acid analog **18**.

### 2.2. Characterization of the Nitroxide Conjugated Compounds by Electron Paramagnetic Resonance (EPR) Spectroscopy

**Figure 2:**
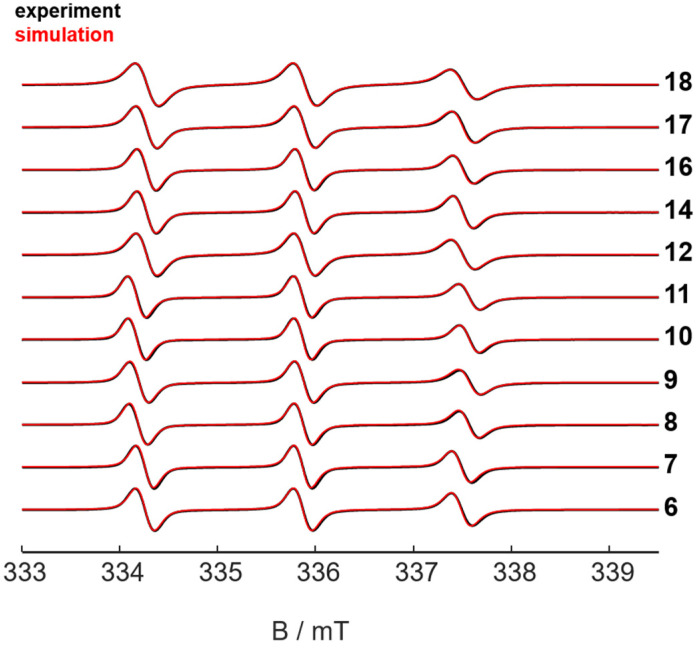
Experimental room temperature CW EPR (X-band, microwave frequency ≈ 9.4 GHz) spectra (black) and the corresponding simulations (red) of the synthesized nitroxide conjugates are given.

The radical nature of the synthesized nitroxide conjugates were verified by continu-ous wave (CW) EPR spectroscopy. Figure 2 shows the CW EPR spectra of the nitroxide conjugated compounds **6**–**12**, **14**, **16**, **17**, and **18**. Conventional triplet pattern of TEMPO nitroxide with relative spectral intensities of 1:1:1 can be seen in Figure 2 due to the coupling of the unpaired electron to the *N*-atom which indicates that the nitroxide was intact during the EPR measurements.

The EPR characteristics, mainly the isotropic hyperfine coupling (A_iso_) and isotropic rotational correlation times ($\tau_{c}$), dominate solution-state EPR spectra as a measure of the line spacing and line shape, were obtained by simulations using the Easyspin software package [28]. All synthesized nitroxide adducts show hyperfine couplings (A_iso_~45–47 MHz) that are indicative of a water-exposed nitroxide moiety. The isotropic rotational correlation times ($\tau_{c}$), as a simple measure of nitroxide rotational dynamics were monitored and were found to be between 1–2 ns for the synthesized nitroxide adducts, in good agreement with what can be expected when attached to medium-sized molecules as in this case. During the simulations, the g-values were kept constant at g_iso_~2.005, the commonly found value for piperidine-based nitroxide radicals [29,30,31]. The numerical values are summarized in Table S1. Altogether, one can state that the EPR parameters clearly show that none of the spin-labelled natural products seems to be aggregated/micellized or non-homogeneously dissolved in aqueous solution.

### 2.3. Cytotoxic Activity

**Figure 3:**
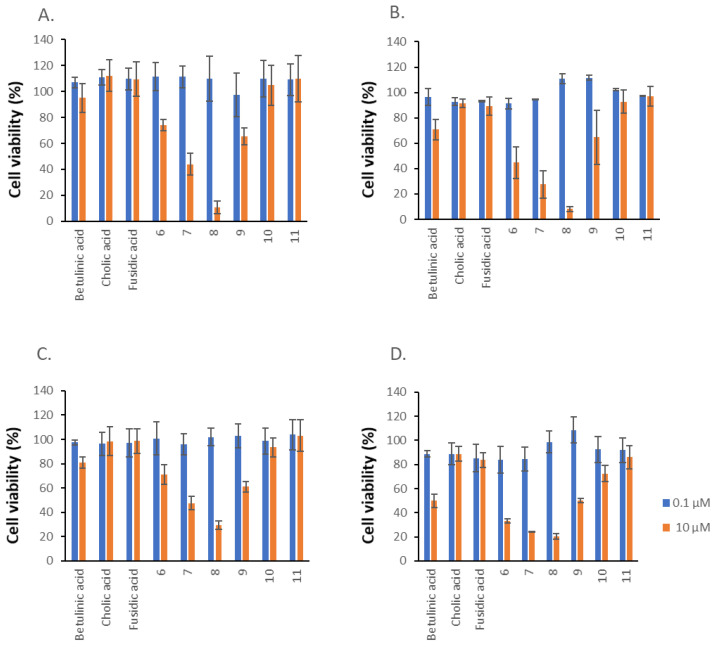
Cell viability of HT29 (**A**,**B**) and PC3 (**C**,**D**); cell lines were treated by the investigated compounds for 48 h. Cell viability was determined using CV assay (**A**,**C**) and MTT assay (**B**,**D**).

The first set of synthesized spin-labelled adducts **6**–**11** were subjected to fast screening by MTT and CV assays to have an overall view on their potential activity against human cancer cell lines viz.: PC3 (prostate cancer) and HT29 (colon cancer). Two concentrations were employed viz.: 0.1 and 10 µM and compared to the activity of unmodified BA (**1**), FA (**2**), and CA (**3**). As shown in Figure 3 both the betulinic acid derivatives **6** and **7** and the fusidic acid derivatives **8** and **9** showed a significant reduction in cell viability when compared to cholic acid derivatives **10** and **11**. The low anticancer activity of cholic acid and its derivatives can be attributed to the high lipophilic character (log p ~ 2.02) and low water solubility, which makes it difficult to cross membranes effectively to be present in high concentrations in the cytosol of the cancer cells [32].

As expected, FA (**2**) failed to show any activity against the tested cancer cell lines. However, the unanticipated very high activity displayed by its derivatives **8** and **9**, especially when compared to the well-known anticancer activity of BA (**1**) was encouraging [33]. The IC_50_ values were also determined for the most active compounds against both cell lines used in our appraisal. The IC_50_ values are illustrated in Table 1, where it is evident that fusidic acid analogue **8** is the most active compound against both cell lines tested. Additionally, another fusidic acid analogue **9** was also active towards HT29 cells with an IC_50_ of 6.98 ± 0.25 μM (CV). Moreover, the betulinic acid analogue **6** possesses cytotoxic effects towards PC3 (IC_50_: 7.43 ± 0.72 μM) and HT29 cells (IC_50_: 8.98 ± 0.43 μM). In addition, it is worthy to note that FA alone possesses no activity when compared to its spin-labelled adducts. Thus, it may clearly be noted that the structure modification provided by the Ugi multicomponent reaction dramatically enhances the anticancer activity of these classes of terpenes.

**Figure 4:**
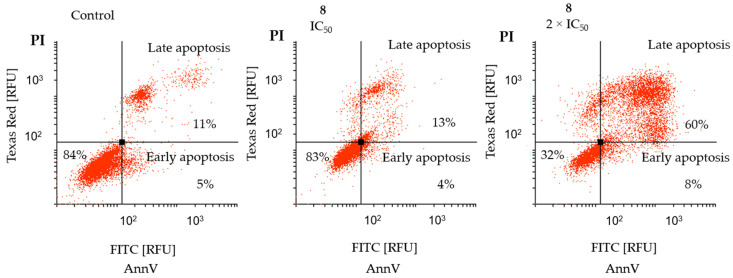
The impact of compound **8** on the apoptosis induction in PC3 cells. **8** was tested using IC_50_, 2 × IC_50_ concentrations for 48 h (AnnV/PI assay).

**Figure 5:**
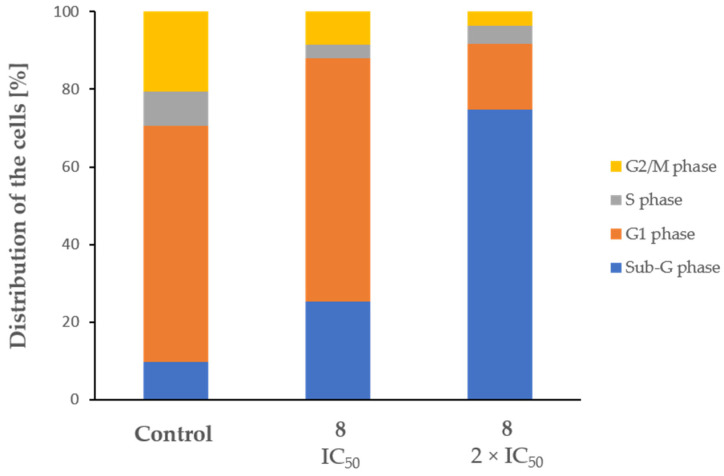
The impact of compound **8** on the distribution of PC3 cells in the cell cycle phases. Compound **8** was tested using IC_50_, 2 × IC_50_ concentrations for 48 h (DAPI assay).

Compound **8**, the most promising conjugate, was selected to determine its mode of action against the PC3 cell line based on its cytotoxic effects. To determine the mode of cell death induced by compound **8**, the AnnV/PI assay was performed since the degree of induction of apoptosis by compound **8** can be effectively measured. The assay determines the expression of phosphatidylserine on the cell surface by annexin V (AnnV) stain and the DNA fragmentation by propidium iodide (PI) (Figure 4). Compound **8** was tested at two different concentrations (IC_50_, 2 × IC_50_) for 48 h and it was analysed using flow cytometry. It is clearly shown, that this compound increases both early and late apoptosis, only when the PC3 cancer cells were treated with 2 × IC_50_ value with a total apoptotic event of 68% compared to the control of 16%. To study the impact of compound **8** on the cell cycle distribution, the DAPI assay was performed as outlined in Figure 5. Based on the results obtained, compound **8** caused a dose-dependent increase in the entrapment of cells in sub G1-phase. This accumulation of the cells in the sub G1-phase of the cell cycle indicates the fragmentation of the DNA that has occurred due to the induction of apoptosis by compound **8**.

**Figure 6:**
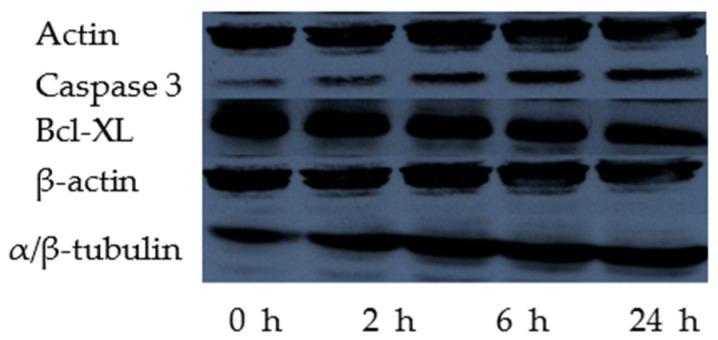
Effect of compound **8** on protein expression in PC3 cells (relative to β-actin).

Reyes et al. reported that natural triterpenoic acids induce caspase-dependent apoptosis and in particular, caspase-3 [34]. Furthermore, recent reports showed that treating tumor cells with nitroxides can also induce apoptosis by a caspase activation mechanism [35]. These studies inspired us to investigate the possibility of caspase-3 being involved in the mechanism of action of conjugate **8** to explain the apoptotic mode of cell death, passivate the level of protein expression of the anti-apoptotic protein Bcl-XL and the housekeeping proteins (β-actin and α/β-tubulin). Results illustrated in Figure 6 show that **8** increased the level of caspase-3 significantly after 48 h of incubation, which clearly indicates the induction of apoptosis by activation of the caspase pathway. Expression of the anti-apoptotic protein Bcl-XL which is a transmembrane molecule in the mitochondria was also measured. After 48 h of incubation, it is clearly evident that the level of Bcl-XL decreases which supports the apoptosis by triggering the caspase-3 activation pathway.

β-Actin is a housekeeping protein that is involved in the restriction of the cell motility, structure integrity, and in addition to its resistance to different cellular treatment, which makes it a good choice as a housekeeping protein for western blot analysis [36]. Indeed, after 48 h of incubation, no change in its expression was detected. α/β-Tubulins is another housekeeping protein control used since their expression should remain unchanged. Surprisingly, the behavior of this protein was manifested by a strong elevation of the expression level being obvious after 48 h of incubation as shown in Figure 6. Recent studies report that microtubulin increases during apoptosis and functions as a physical barrier preventing caspase from spreading into the cellular cortex. In addition, it increases phosphatidylserine (PS) externalization which helps the macrophage for efficient clearance [37].

The influence of compound **8** (IC_50_ and 2 × IC_50_) on the ROS production in PC3 cells was monitored using dihydrorhodamine (DHR) assay for 48 h and the data were analysed with flow cytometry. 

**Figure 7:**
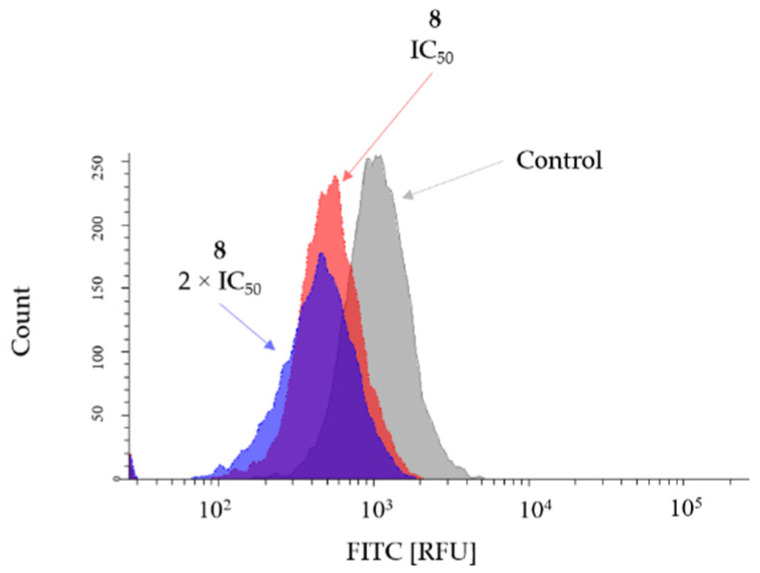
The impact of compound **8** on ROS produced by PC3. **8** was tested using IC_50_, 2 × IC_50_ concentrations for 48 h using DHR assay, fluorescence was detected in FITC channel (ex/em: 488/520 nm).

As shown in Figure 7 compound **8** indeed reduced the level of ROS as anticipated in a dose-dependent manner.

### 2.4. Fluorescent Imaging Study

**Figure 8:**
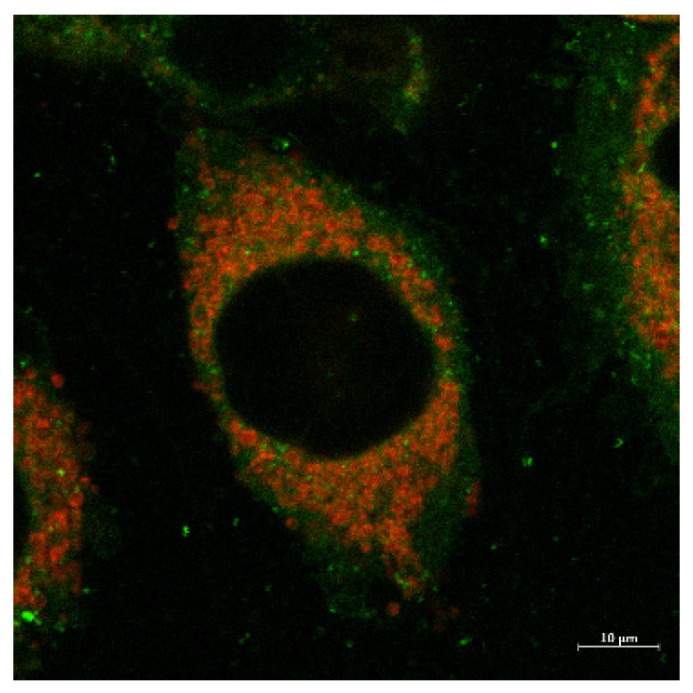
Fluorescent imaging with fluorescent conjugate **12** (ex/em: 488/520 nm) double-stained with MitoTracker^TM^ Deep Red (ex/em: 596/615 nm) in PC3 cancer cell line.

**Figure 9:**
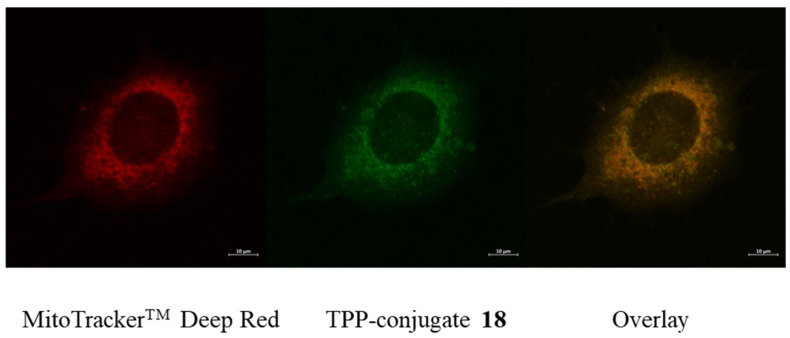
Fluorescent imaging of PC3 cancer cell line stained with TPP-conjugate **18** (ex/em:488/520 nm) and with MitoTracker^TM^ Deep Red (ex/em: 596/615 nm).

Fluorescent conjugate **12** was initially used to determine if this dye-tagged analogue of fusidic acid conjugate **8** can target the mitochondria, since mitochondria are the major source of ROS generation and therefore is more sensible for ROS manipulation. Unfortunately, after PC3 cancer cells were incubated with **12** (depicted as green color in Figure 8), no mitochondrial targeting was observed. Therefore, we turned our attention to test the mitochondrial targeting of TPP-conjugate **18**. After 24 h of incubation of compound **18** with PC3 cells, a clear mitochondrial targeting was successfully achieved as shown in Figure 9. Additionally, cytotoxic activity of **18** against PC3 and HT29 was found to be with significant effects towards PC3 cancer cell line (IC_50_: 6.18 ± 0.20 μM, MTT).

## 3. Conclusions

TEMPO radical conjugation to natural products can serve as a potential strategy to obtain new hybrid compounds with novel mechanisms of action. Conjugate **8** revealed a high activity against both PC3 and HT29 cancer cell lines (PC3: IC_50_ 6.0 ± 1.1 μM; HT29: IC_50_ 7.4 ± 0.6 μM), furthermore, apoptosis was induced through the caspase activation mechanism. In addition, targeting mitochondria (the major source of ROS generation) was successfully achieved with **18**. Moreover, it was clearly demonstrated that utilizing Ugi multicomponent reactions is a powerful synthetic tool that gives access to a wide variety of different analogues via a fairly easy synthetic effort. We envisioned that utilizing the power of MCR, large interesting libraries of natural product TEMPO conjugates for the treatment of cancer can be generated.

## 4. Materials and Methods

### 4.1. Chemistry

#### 4.1.1. Materials

All commercially available reagents were purchased and used without further purification. Convertible isocyanide 2-isocyano-2-methylpropyl phenyl carbonate “IPB” was synthesized following reported procedures [27]. (3-Aminopropyl)triphenylphosphonium bromide (TPP-NH_2_) was also synthesized following reported protocols [38]. HPLC grade methanol was used in all Ugi reactions. Analytical thin layer chromatography (TLC) was performed using silica gel 60 F_254_ aluminum sheets (Merck, Darmstadt, Germany) and the visualization of the spots has been done under UV light (254 nm) or by developing with a solution of cerium sulfate. Flash column chromatography was performed using silica gel (0.040–0.063 mm). ^1^H- and ^13^C-NMR spectra were recorded in solutions on a 400 NMR Varian MERCURY-VX 400 at 22 °C at 400 MHz and 100 MHz, or an Agilent (Varian, Santa Clara, CA, USA) VNMRS 600 NMR spectrometer at 599.83 MHz and 150.83 MHz respectively. Chemical shifts (δ) are reported in ppm relative to TMS (^1^H-NMR) and to the solvent signal (^13^C NMR spectra). The positive-ion high-resolution ESI mass spectra were obtained with an Orbitrap Elite mass spectrometer (Thermo Fisher Scientific, Schwerte, Germany) equipped with HESI electrospray ion source (positive spray voltage 4 kV, capillary temperature 275 °C, source heater temperature 80 °C, FTMS resolution 60,000). Nitrogen was used as sheath gas. The instrument was externally calibrated using the Pierce LTQ Velos ESI positive ion calibration solution (product number 88323, ThermoFisher Scientific, Rockford, IL, USA). The data were evaluated using the software Xcalibur 2.7 SP1. Analytical RP-HPLC analysis was performed with an 1100 system (Agilent, Santa Clara, CA, USA) on a reverse-phase C18 column (4.6 × 150 mm, 5 μm) with a PDA detector. A linear gradient from 5% to 100% of solvent B in solvent A over 15–30 min at a flow rate of 0.8 mL min^−1^. Detection was accomplished at 210 nm. Solvent A: 0.1% (*v/v*) formic acid (FA) in water. Solvent B: 0.1% (*v/v*) FA in acetonitrile.

PBS, RPMI 1640, and Trypsin EDTA were from Capricorn Scientific (Ebsdorfergrund, Germany). β-mercaptoethanol was from Bio-Rad (Hercules, CA, USA). Anti-rabbit IgG HRP-linked antibody, α/β-tubulin rabbit Ab, caspase-3 rabbit Ab were purchased from Cell Signalling Technology (Frankfurt/Main, Germany), while Bcl-XL rabbit antibody was obtained from Abcam (Cambridge, UK). DMSO was bought from Duchefa Biochemie (Harleem, The Netherlands). ECL Prime Western Blotting System was supplied by GE Healthcare (Braunschweig, Germany). AnnV/PI, PAGE Ruler, EDTA Solution, Trypan blue, MitoTracker^TM^ Deep Red and Halt Protease Inhibitor Cocktail were obtained from ThermoFisher Scientific, Schwerte, Germany). Ethanol, Na_2_HPO_4_, NaH_2_PO_4_, and BSA were bought from Merck, Darmstadt, Germany). Digitonin was from Riedel De Haen (Seelze, Germany). Acetic acid, APS, FCS, glycerol, glycine, methanol, NaOH, penicillin/streptomycin, Roti-quant “5x”, TEMED and TRIS were from Roth (Karlsruhe, Germany). Acrylamide/Bisacrylamide was bought from Serva (Heidelberg, Germany). Finally, bromophenol blue, CV, DAPI, MTT, Triton X-100, and Tween-20 were from Sigma Aldrich (St. Louis, MO, USA).

#### 4.1.2. General Procedure A for the Ugi-4CR

To a stirred solution of TEMPO amine **4** (0.1 mmol) in methanol (250 µL, 0.4 M) was added paraformaldehyde (0.1 mmol) and the mixture was stirred for 2 h. After this time the acid (0.1 mmol) and isonitrile (0.1 mmol) were added before stirring was continued for 18 h. The solvent was removed under reduced pressure and the crude material purified by column chromatography to afford the desired products.

Note: due to the paramagnetism of nitroxide moieties, NMR cannot provide information useful for structural elucidation of nitroxide-containing products, therefore, reduction of the paramagnetic center was performed with phenylhydrazine or hydrazobenzene [39,40].

*(4-{[2-(tert-Butylamino)-2-oxoethyl][(1R,3aS,5aR,5bR,9S,11aR)-9-hydroxy-5a,5b,8,8,11a-pentamethyl-1-(prop-1-en-2-yl)icosahydro-3aH-cyclopenta[a]chrysene-3a-carbonyl]amino}-2,2,6,6-tetramethylpiperidin-1-yl)oxidanyl* (**6**)

Obtained using the general method **A**, *N*-oxyl amine **4** and paraformaldehyde were used, then betulinic acid and *t*-butyl isonitrile were added. The crude reaction product was purified by silica gel column chromatography (ethyl acetate/hexane 8:2) to yield compound **6** (51 mg, 0.070 mmol, 70%) as red solid. *R_F_* 0.77 (ethyl acetate/hexane 8:2). NMR of the corresponding hydroxylamine after phenylhydrazine reduction. ^1^H-NMR (600 MHz, CDCl_3_) δ 4.72 (s, 1H), 4.59 (s, 1H), 4.35 (d, *J* = 12.6 Hz, 1H), 3.65 (s, 2H), 3.16 (dd, *J* = 11.3, 4.8 Hz, 2H), 2.98 (m, 1H), 2.87–2.81 (m, 1H), 2.24–2.18 (m, 1H), 2.06–1.20 (m, 49H), 0.96 (s, 3H), 0.95 (s, 3H), 0.92 (s, 3H), 0.80 (s, 3H), 0.74 (s, 3H). ^13^C-NMR (151 MHz, CDCl_3_): δ 176.0 (CO), 109.5, 78.9, 55.5, 54.9, 53.0, 50.9, 50.8, 49.3, 47.7, 45.8, 42.8, 42.3, 42.1, 40.8, 38.9, 38.8, 37.3, 36.9, 36.1, 34.3, 32.2, 32.0, 31.6, 29.9, 29.8, 28.7 (4 × CH_3_ (TEMPO)), 28.1 (CH_3_ (*t*Bu)), 27.5, 25.7, 21.1, 20.1, 19.9, 19.6, 18.3, 16.2, 15.5, 14.8. HRMS (ESI) *m/z*: 723.5890 [M + H]^+^, calcd. for [C_45_H_77_N_3_O_4_]^+^ 723.5914.

*[4-([(1R,3aS,5aR,5bR,9S,11aR)-9-Hydroxy-5a,5b,8,8,11a-pentamethyl-1-(prop-1-en-2-yl)icosahydro-3aH-cyclopenta[a]chrysene-3a-carbonyl]{2-oxo-2-[(2,4,4-trimethoxybutyl)amino]ethyl}-amino)-2,2,6,6-tetramethylpiperidin-1-yl]oxidanyl* (**7**)

Obtained using the general method **A**, *N*-oxyl amine **4** and paraformaldehyde were used, then betulinic acid and IPB isonitrile **5** were added. The crude reaction product was purified by silica gel column chromatography (ethyl acetate/hexane 8:2) to yield compound **7** (50 mg, 0.061 mmol, 61%) as red solid. *R_F_* 0.35 (ethyl acetate/hexane 8:2). NMR of the corresponding hydroxylamine after phenylhydrazine reduction. ^1^H-NMR (600 MHz, CDCl_3_) δ 4.72 (s, 1H), 4.59 (s, 1H), 4.54–4.49 (m, 1H). 4.38 (t, *J* = 12.5 Hz, 1H (ipb)), 3.66 (s, 2H), 3.36–3.30 (m, 12H). (IPB Ugi moiety), 3.16 (dd, *J* = 11.3, 4.8 Hz, 2H), 2.98 (m, 1H), 2.87–2.81 (m, 1H), 2.27–2.15 (m, 1H), 2.11–1.09 (m, 42H), 0.96 (s, 3H), 0.95 (s, 3H), 0.91 (s, Hz, 3H), 0.81 (s, 3H), 0.75 (s, 3H). ^13^C-NMR (151 MHz, CDCl_3_) δ 176.08 (CO), 109.50, 79.08, 57.04, 56.98, 56.49, 55.57, 55.47, 53.24, 53.07, 50.93, 45.82, 45.77, 42.59, 42.09, 41.51, 41.30, 40.86, 38.99, 38.85, 38.37, 37.34, 36.10, 35.20, 34.39, 32.21, 31.61, 31.52, 31.44, 31.01, 29.97, 29.82, 28.11(4 x CH_3_ (TEMPO)), 27.53, 25.73, 21.21, 21.17, 20.32, 20.17, 19.71, 16.31, 16.08, 16.05, 15.48, 14.83. HRMS (ESI) *m/z*: 812.6138 [M]^+^, calcd. for [C_48_H_82_N_3_O_7_]^+^ 812.6153.

*[4-({(2E)-2-[(2S,3aS,3bS,6S,7R,9aS,10R,11aR)-2-(Acetyloxy)-7,10-dihydroxy-3a,3b,6,9a-tetramethylhexadecahydro-1H-cyclopenta[a]phenanthren-1-ylidene]-6-methylhept-5-enoyl}[2-(tert-butylamino)-2-oxoethyl]amino)-2,2,6,6-tetramethylpiperidin-1-yl]oxidanyl* (**8**)

Obtained using the general method **A**, *N*-oxyl amine **4** and paraformaldehyde were used, then fusidic acid and *t*-butyl isonitrile were added. The crude reaction product was purified by silica gel column chromatography (DCM/MeOH 9:1) to yield compound **8** (51 mg, 0.057 mmol, 57%) as red solid. *R_F_* 0.65 (DCM/MeOH 9:1). NMR of the corresponding hydroxylamine after phenylhydrazine reduction. ^1^H-NMR (600 MHz, CDCl_3_) δ 5.69 (d, *J* = 8.6 Hz, 1H), 5.07 (m, 1H), 4.33–4.22 (m, 3H), 3.71 (s, 2H), 3.26 (d, *J* = 14.6 Hz, 1H), 3.05–3.02 (m, 1H), 2.80–2.75 (m, 2H), 2.32–2.28 (m, 1H), 2.22–2.02 (m, 5H), 1.89 (s, 3H), 1.86–1.82 (m, 2H), 1.76–1.71 (m, 4H), 1.68 (s, 3H), 1.61 (s, 3H), 1.60–1.53 (m, 4H), 1.34 (d, *J* = 7.0 Hz, 3H), 1.28 (s, 9H), 1.26–1.14 (m, 15H), 1.13–1.08 (m, 2H), 0.96 (s, 3H), 0.91–0.88 (m, 6H). ^13^C-NMR (151 MHz, CDCl_3_) δ 173.21, 169.87, 169.06, 133.15, 132.45, 122.56, 74.81, 73.10, 71.29, 68.10, 50.95, 50.84, 49.76, 49.63, 49.42, 44.51, 41.63, 39.45, 39.26, 37.09, 36.22, 36.09, 35.22, 32.28, 32.28, 30.28, 30.24, 28.69, 28.57 (4 x CH_3_ (TEMPO)), 28.52, 25.86, 22.97, 20.86, 20.51, 20.10, 18.05, 17.88, 16.03. HRMS (ESI) *m/z*: 783.5744 [M + H]^+^, calcd. for [C_46_H_77_N_3_O_7_]^+^ 783.5762.

*[4-({(2E)-2-[(2S,3aS,3bS,6S,7R,9aS,10R,11aR)-2-(Acetyloxy)-7,10-dihydroxy-3a,3b,6,9a-tetramethylhexadecahydro-1H-cyclopenta[a]phenanthren-1-ylidene]-6-methylhept-5-enoyl}{2-oxo-2-[(2,4,4-trimethoxybutyl)amino]ethyl}amino)-2,2,6,6-tetramethylpiperidin-1-yl]oxidanyl* (**9**)

Obtained using the general method **A**, *N*-oxyl amine **4** and paraformaldehyde were used, then fusidic acid and IPB isonitrile **5** were added. The crude reaction product was purified by silica gel column chromatography (DCM/MeOH 9:1) to yield compound **9** (60 mg, 0.068 mmol, 69%) as red solid. *R_F_* 0.72 (DCM/MeOH 9:1). NMR of the corresponding hydroxylamine after phenylhydrazine reduction. ^1^H-NMR (600 MHz, CDCl_3_) δ 5.69 (d, *J* = 8.4 Hz, 1H), 5.08 (m, 1H), 4.51 (m, 1H), 4.34–4.31 (m, 1H), 3.76–3.73 (m, 1H), 3.65 (s, 2H), 3.39–3.29 (m, 12H), 3.14 (m, 1H), 3.07–3.02 (m, 1H), 2.75 (m, 1H), 2.37–2.27 (m, 1H), 2.23–2.08 (m, 5H), 2.04 (s, 3H), 1.92 (s, 3H), 1.88–1.80 (m, 4H), 1.76–1.71 (m, 4H), 1.68 (s, 3H), 1.62 (s, 3H), 1.60–1.48 (m, 4H), 1.36 (s, 3H), 1.31–1.17 (m, 15H), 1.15–1.10 (m, 2H), 0.97 (s, 3H), 0.93–0.88 (m, 6H). ^13^C-NMR (151 MHz, CDCl_3_) δ 173.21, 169.87, 169.06, 133.15, 132.45, 122.56, 74.81, 71.29, 68.10, 50.95, 49.63, 49.42, 44.51, 42.68, 39.45, 39.26, 37.09, 36.37, 36.22, 36.09, 35.22, 32.28, 30.28, 30.24, 30.07, 28.57 (4 x CH_3_ (TEMPO)), 28.52, 27.81, 27.73, 25.86, 23.85, 22.97, 21.43, 21.21, 20.86, 20.51, 20.10, 18.19, 18.05, 17.88, 16.03.15.68. HRMS (ESI) *m/z*: 873.6062 [M + H]^+^, calcd. for [C_49_H_83_N_3_O_10_]^+^ 873.6078.

*[4-([2-(tert-Butylamino)-2-oxoethyl]{(4R)-4-[(1R,3aS,4R,7R,9aS,9bS,11S,11aR)-4,7,11-trihydroxy-9a,11a-dimethylhexadecahydro-1H-cyclopenta[a]phenanthren-1-yl]pentanoyl}amino)-2,2,6,6-tetramethylpiperidin-1-yl]oxidanyl* (**10**)

Obtained using the general method **A**, *N*-oxyl amine **4** and paraformaldehyde were used, then cholic acid and *t*-butyl isonitrile were added. The crude reaction product was purified by silica gel column chromatography (DCM/MeOH 9:1) to yield compound **10** (55 mg, 0.081 mmol, 81%) as red solid. *R_F_* 0.62 (DCM/MeOH 9:1). NMR of the corresponding hydroxylamine after phenylhydrazine reduction. ^1^H-NMR (600 MHz, CDCl_3_) δ 4.14–4.08 (m, 1H), 4.06–3.98 (m, 2H), 3.97–3.91 (m, 1H), 3.85–3.75 (m, 1H), 2.51–2.42 (m, 1H), 2.36–2.28 (m, 1H), 2.27–2.13 (m, 4H), 1.94–1.80 (m, 5H), 1.79–1.60 (m, 7H), 1.61–1.46 (m, 7H), 1.37–1.15 (m, 25H), 1.13–1.00 (m, 3H), 0.97 (s, 3H), 0.87 (s, 3H), 0.67 (d, *J* = 7.5 Hz, 3H). ^13^C-NMR (151 MHz, CDCl_3_) δ 173.21, 169.87, 169.06, 133.15, 132.45, 122.56, 74.81, 73.10, 71.29, 68.10, 50.95, 50.84, 49.76, 49.63, 49.42, 44.51, 41.63, 39.45, 39.26, 37.09, 36.22, 36.09, 35.22, 32.28, 32.28, 30.28, 30.24, 28.57, 28.52, 28.52, 25.86, 22.97, 20.86, 20.51, 20.10, 18.05, 17.88, 16.03. HRMS (ESI) *m/z*: 675.5164 [M + H]^+^, calcd. for [C_39_H_69_N_3_O_6_]^+^ 675.5186.

*[2,2,6,6-Tetramethyl-4-({2-oxo-2-[(2,4,4-trimethoxybutyl)amino]ethyl}{(4R)-4-[(1R,3aS,4R,7R,9aS,9bS,11S,11aR)-4,7,11-trihydroxy-9a,11a-dimethylhexadecahydro-1H-cyclopenta[a]phenanthren-1-yl]pentanoyl}amino)piperidin-1-yl]oxidanyl* (**11**)

Obtained using the general method **A**, *N*-oxyl amine **4** and paraformaldehyde were used, then cholic acid and IPB isonitrile **5** were added. The crude reaction product was purified by silica gel column chromatography (DCM/MeOH 9:1) to yield compound **11** (47 mg, 0.061 mmol, 61%) as red solid. *R_F_* 0.55 (DCM/MeOH 9:1). NMR of the corresponding hydroxylamine after phenylhydrazine reduction. ^1^H-NMR (600 MHz, CDCl_3_) δ 4.55–4.48 (m, 1H), 4.12–4.00 (m, 1H), 3.95–3.81 (m, 2H), 3.45 (s, 3H), 3.37–3.28 (m, 12H), 3.28–3.22 (m, 1H), 2.48 (m, 2H), 2.36–2.11 (m, 5H), 1.98–1.80 (m, 6H), 1.80–1.69 (m, 5H), 1.69–1.39 (m, 7H), 1.40–1.16 (m, 15H), 1.15–1.00 (m, 4H), 0.96 (s, 3H), 0.88 (s, 3H), 0.68 (s, 3H). ^13^C-NMR (151 MHz, CDCl_3_) δ 174.58, 170.07, 151.16, 101.75, 76.18, 72.86, 71.70, 68.28, 59.33, 59.28, 57.03, 53.33, 53.06, 52.96, 50.55, 46.99, 46.39, 45.61, 41.80, 41.38, 41.28, 35.57, 35.20, 35.09, 34.64, 32.16, 31.54, 30.85, 30.39, 28.20, 27.46, 26.51, 23.12, 22.42, 19.88, 17.44, 14.62, 12.48. HRMS (ESI) *m/z*: 764.5410 [M]^+^, calcd. for [C_42_H_74_N_3_O_9_]^+^ 764.5425.

*[4-({(2E)-2-[(2S,3aS,3bS,6S,7R,9aS,10R,11aR)-2-(Acetyloxy)-7,10-dihydroxy-3a,3b,6,9a-tetramethylhexadecahydro-1H-cyclopenta[a]phenanthren-1-ylidene]-6-methylhept-5-enoyl}[2-({2-[6-(dimethylamino)-1,3-dioxo-1H-benzo[de]isoquinolin-2(3H)-yl]ethyl}amino)-2-oxoethyl]amino)-2,2,6,6-tetramethylpiperidin-1-yl]oxidanyl* (**12**)

Obtained using the general method **A**, *N*-oxyl amine **4** and paraformaldehyde were used, then fusidic acid and Yudin’s isonitrile were added. The crude reaction product was purified by silica gel column chromatography (DCM/MeOH 9:1) to yield compound **12** (35 mg, 0.035 mmol, 35%) as yellow powder. *R_F_* 0.1 (DCM/MeOH 9:1). NMR of the corresponding hydroxylamine after the addition of hydrazobenzene. ^1^H-NMR (400 MHz, DMSO-*d*_6_) δ 8.49 (dd, *J* = 8.5, 2.1 Hz, 1H), 8.45 (d, *J* = 7.5 Hz, 1H), 8.34 (dd, *J* = 8.2, 1.9 Hz, 1H), 7.78–7.72 (m, 1H), 7.20 (d, *J* = 8.6 Hz, 1H), 5.65 (d, *J* = 8.5 Hz, 1H), 5.09–5.01 (m, 1H), 4.91–4.76 (m, 2H), 4.18–4.11 (m, 1H), 4.07 (d, *J* = 3.9 Hz, 1H), 3.99 (d, *J* = 3.9 Hz, 1H), 3.90–3.81 (m, 2H), 3.53 (s, 2H), 3.50–3.47 (m, 1H), 3.07 (s, 6H), 2.99–2.85 (m, 1H), 2.70–2.60 (m, 1H), 2.40–2.17 (m, 3H), 2.16–1.93 (m, 5H), 1.85 (d, *J* = 13.3 Hz, 2H), 1.62 (dd, *J* = 9.8, 5.2 Hz, 7H, (Fusidic acid; 5H+TEMPO; 2H)), 1.57–1.50 (m, 6H), 1.49–1.28 (m, 6H), 1.25 (s, 3H), 1.08–0.95 (m, 17H, (fusidic acid 3H, TEMPO 14H)), 0.86 (s, 3H), 0.81–0.75 (m, 6H). ^13^C-NMR (101 MHz, DMSO-*d_6_*) δ 171.61, 169.91, 169.10, 164.32, 163.64, 147.18, 133.72, 132.31, 131.95, 131.88, 130.94, 130.21, 125.46, 124.76, 123.96, 123.48, 114.04, 113.47, 74.34, 69.68, 66.23, 58.57, 58.38, 51.63, 49.42, 49.07, 48.98, 48.65, 44.84, 43.22, 39.05, 36.90, 36.81, 35.67, 35.55, 33.31, 33.05, 32.37, 32.25, 30.69, 29.93, 28.82, 28.49, 25.92, 23.94, 23.18, 20.67, 20.13, 18.01, 17.97, 16.74, 16.71. HRMS (ESI) *m/z*: 993.6145 [M + H]^+^, calcd. for [C_58_H_83_N_5_O_9_]^+^ 993.6191.

#### 4.1.3. General Procedure **B** for the Conversion of Ugi Products 7 and 9 to Corresponding Spin-Labelled N-Acylpyrroles **13** and **15**

To a solution of Ugi products **7** and **9** (0.05 mmol) in toluene (10 mL) was added 10-camphorsulfonic acid (10 mol%) and quinoline (10 mol%). The mixture was stirred for 1 min at room temperature and then refluxed for at least 30 min until TLC showed complete conversion. The mixture was cooled to room temperature, transferred to a separatory funnel and washed with 1M aqueous HCl (2 × 30 mL). The acidic aqueous phase was further extracted with ethyl acetate (1 × 20 mL). The organic layers were combined, washed with NaHCO_3_ and brine (2 × 20 mL), dried over anhydrous Na_2_SO_4_, filtered and evaporated under reduced pressure to obtain the *N*-acyl pyrrole derivatives **13** and **15**, which were used in the next step without further purification.

#### 4.1.4. General Procedure **C** for the Conversion of the N-Acylpyrroles **13** and **15** into Their Corresponding Carboxylic Acids **14** and **16**

##### Method **C1**

To a solution of intermediates **13** and **15** (0.025 mmol) in a mixture of THF (2 mL), methanol (2 mL), water (2 mL), potassium hydroxide (0.5 mmol) was added. This mixture was heated to 110 °C for 30 min in a microwave (90 W heating, 6 W keeping temperature). The reaction mixture was diluted with methanol (10 mL) and the pH value was set to pH = 2 by the addition of saturated aqueous NaHSO_4_ solution. The aqueous phase was extracted with ethyl acetate (3 × 100 mL) and it was dried over Na_2_SO_4_. After filtration and evaporation of the solvent, the crude residue was purified by column chromatography (DCM/MeOH 8:2). By this method compounds, **14** and **16** were obtained.

*(4-{(Carboxymethyl)[(1R,3aS,5aR,5bR,9S,11aR)-9-hydroxy-5a,5b,8,8,11a-pentamethyl-1-(prop-1-en-2-yl)icosahydro-3aH-cyclopenta[a]chrysene-3a-carbonyl]amino}-2,2,6,6-tetramethylpiperidin-1-yl)oxidanyl* (**14**)

Obtained using the general method **C1**, the reaction crude reaction product was purified by silica gel column chromatography (DCM/MeOH 8:2) to yield compound **14** (11.8 mg, 0.014 mmol, 70%) as orange powder. *R_F_* 0.12 (DCM/MeOH 8:2). NMR of the corresponding hydroxylamine after the addition of hydrazobenzene. ^1^H-NMR (400 MHz, DMSO-*d_6_*) δ 11.92 (s, 1H), 4.64 (d, *J* = 2.7 Hz, 1H), 4.57–4.51 (m, 1H), 4.26 (d, *J* = 5.1 Hz, 1H), 3.80–3.62 (m, 1H), 3.52 (s, 2H), 2.97 (m, 2H), 2.80 (m, 1H), 2.68 (m, 1H), 2.36–2.31 (m, 1H), 1.96 (m, 2H), 1.78 (dd, *J* = 13.0, 9.0 Hz, 2H), 1.64 (s, 3H), 1.55 (t, *J* = 12.7 Hz, 5H), 1.51–1.38 (m, 6H), 1.38–1.21 (m, 8H), 1.08 (d, *J* = 3.1 Hz, 14H), 0.92 (s, 3H), 0.88 (s, 3H), 0.84 (s, 3H), 0.77 (s, 3H), 0.66 (s, 3H). ^13^C-NMR (101 MHz, DMSO-*d_6_*) δ 173.71, 171.03, 151.04, 109.22, 76.75, 58.20, 54.97, 53.73, 52.04, 50.17, 48.12, 45.40, 43.82, 41.50, 38.48, 38.28, 38.23, 36.73, 35.18, 33.92, 32.46, 30.63, 29.07, 27.15, 27.12, 25.15, 20.65, 19.78, 19.63, 19.07, 17.93, 15.97, 15.85, 15.78, 14.31. HRMS (ESI) *m/z*: 666.4996 [M − H]^−^, calcd. for [C_41_H_66_N_2_O_5_]^−^ 666.4972.

*{4-[(Carboxymethyl){(2E)-6-methyl-2-[(2S,3aS,3bS,6S,7R,9aS,10R,11aR)-2,7,10-trihydroxy-3a,3b,6,9a-tetramethylhexadecahydro-1H-cyclopenta[a]phenanthren-1-ylidene]hept-5-enoyl}-amino]-2,2,6,6-tetramethylpiperidin-1-yl}oxidanyl* (**16**)

Obtained using the general method **C1**, the reaction crude reaction product was purified by silica gel column chromatography (DCM/MeOH 8:2) to yield compound **16** (10 mg, 0.014 mmol, 58%) as orange oil. *R_F_* 0.10 (DCM/MeOH 8:2). NMR of the corresponding hydroxylamine after the additon of hydrazobenzene. ^1^H-NMR (400 MHz, DMSO-*d_6_*) δ 10.22 (s, 1H), 5.46 (d, *J* = 8.6 Hz, 1H), 5.14–5.05 (m, 1H), 4.48 (d, *J* = 8.3 Hz, 1H), 4.17–4.07 (m, 1H), 4.00 (d, *J* = 3.6 Hz, 1H), 3.94 (d, *J* = 3.9 Hz, 1H), 3.51 (t, *J* = 3.2 Hz, 1H), 3.17 (dd, *J* = 15.1, 9.9 Hz, 1H), 2.99–2.75 (m, 1H), 2.71–2.55 (m, 1H), 2.40–1.93 (m, 8H), 1.93–1.66 (m, 4H), 1.63 (s, 3H), 1.55 (t, *J* = 2.2 Hz, 3H), 1.52–1.29 (m, 6H), 1.26 (s, 3H), 1.24–1.07 (m, 3H), 1.07–0.91 (m, 14H), 0.89 (d, *J* = 6.2 Hz, 6H), 0.79 (d, *J* = 6.7 Hz, 3H). ^13^C-NMR (101 MHz, DMSO-*d_6_*) δ 174.42, 171.52, 147.95, 147.48, 135.99, 124.70, 69.98, 69.76, 66.50, 58.62, 58.55, 56.47, 49.67, 49.07, 48.89, 43.28, 41.71, 39.22, 36.97, 35.86, 35.67, 33.37, 33.13, 30.75, 29.57, 27.60, 25.89, 23.95, 23.06, 21.21, 19.01, 18.17, 16.77, 16.00. HRMS (ESI) *m/z*: 684.4732 [M − H]^−^, calcd. for [C_40_H_64_N_2_O_7_]^−^ 684.4714.

##### Method **C2**

Method **C2** was established to keep the C-16 acetyl group intact on position 16 of the fusidic acid skeleton. *N*-acylpyrrole **15** (0.025 mmol) was dissolved in a mixture of *t*-BuOH (10 mL) and H_2_O (5 mL). Then, DMAP (0.015 mmol) was added and the reaction mixture was heated at reflux for 5 h, after which TLC (DCM/MeOH 8:2) indicated the saponification into the carboxylic acid **17**. The reaction mixture was concentrated to a volume of 10 mL in a rotary evaporator. Saturated NaHCO_3_ solution (10 mL) and CH_2_Cl_2_ (20 mL) were added. After the separation of the organic layer, the water layer was extracted with CH_2_Cl_2_ (2 × 30 mL). Then the water layer was acidified with NaHSO_4_ (2 M) and extracted with EtOAc (3 × 20 mL). The combined organic solutions of the acidic extraction were dried over Na_2_SO_4_, filtered, and evaporated to give carboxylic acid derivative, which was further purified by column chromatography (DCM/MeOH 8:2).

*{4-[{(2E)-2-[(2S,3aS,3bS,6S,7R,9aS,10R,11aR)-2-(Acetyloxy)-7,10-dihydroxy-3a,3b,6,9a-tetramethylhexadecahydro-1H-cyclopenta[a]phenanthren-1-ylidene]-6-methylhept-5-enoyl}(carboxymethyl)amino]-2,2,6,6-tetramethylpiperidin-1-yl}oxidanyl* (**17**)

Obtained using the general method **C2**, the reaction crude reaction product was purified by silica gel column chromatography (DCM/MeOH 8:2) to yield compound **17** (12 mg, 0.016 mmol, 66%) as orange oil. *R_F_* 0.15 (DCM/MeOH 8:2). NMR of the corresponding hydroxylamine after the addition of hydrazobenzene. ^1^H-NMR (400 MHz, DMSO-*d_6_*) δ 8.89 (s, 1H), 5.44 (d, *J* = 8.5 Hz, 1H), 5.10 (t, *J* = 7.0 Hz, 1H), 4.18–4.13 (m, 1H), 3.98 (d, *J* = 3.7 Hz, 1H), 3.95 (d, 1H), 3.87 (d, *J* = 17.0 Hz, 2H), 3.53–3.49 (m, 1H), 2.97–2.87 (m, 1H), 2.73–2.66 (m, 1H, TEMPO), 2.26–2.17 (m, 3H), 2.10–1.97 (m, 5H), 1.94–1.82 (m, 5H), 1.81–1.70 (m, 2H, TEMPO), 1.64 (s, 3H), 1.57 (s, 3H), 1.53–1.29 (m, 6H), 1.27 (s, 3H), 1.26–1.12 (m, 3H), 1.10–0.95 (m, 14H, TEMPO), 0.88 (s, 3H), 0.84 (s, 3H), 0.79 (d, *J* = 6.7 Hz, 3H). ^13^C-NMR (101 MHz, DMSO-*d_6_*) δ 175.94, 171.54, 170.02, 154.96, 147.92, 133.53, 123.91, 74.27, 69.74, 66.27, 58.63, 58.47, 54.20, 49.43, 49.10, 48.97, 43.02, 39.25, 38.90, 36.94, 36.69, 36.09, 35.71, 31.77, 29.78, 27.52, 26.07, 23.86, 23.22, 21.37, 20.72, 20.18, 18.21, 18.15, 16.78, 14.43. HRMS (ESI) *m/z*: 726.4807 [M − H]^−^, calcd. for [C_42_H_66_N_2_O_8_]^−^ 726.4819.

*{4-[{(2E)-2-[(2S,3aS,3bS,6S,7R,9aS,10R,11aR)-2-(Acetyloxy)-7,10-dihydroxy-3a,3b,6,9a-tetramethylhexadecahydro-1H-cyclopenta[a]phenanthren-1-ylidene]-6-methylhept-5-enoyl}(2-{[2-({2-[6-(dimethylamino)-1,3-dioxo-1H-benzo[de]isoquinolin-2(3H)-yl]ethyl}amino)-2-oxoethyl][3-(triphenylphosphaniumyl)propyl]amino}-2-oxoethyl)amino]-2,2,6,6-tetramethylpiperidin-1-yl}oxidanyl bromide* (**18**)

Obtained using the general method **A** in 0.013 mmol scale (3-aminopropyl) triphenylphosphonium bromide and paraformaldehyde were used, then fusidic acid derivative **17** and Yudin’s isonitrile were added. The crude reaction product was purified by silica gel column chromatography (DCM/MeOH 8:2) to yield compound **18** (6 mg, 0.004 mmol, 34%) as yellow powder. *R_F_* 0.12 (DCM/MeOH 8:2). NMR of the corresponding hydroxylamine after the additon of hydrazobenzene. ^1^H-NMR (400 MHz, DMSO-*d_6_*) δ 8.57 (d, *J* = 8.8 Hz, 1H), 8.54 (d, *J* = 7.5 Hz, 1H), 8.51 (d, *J* = 8.3 Hz, 1H), 7.87–7.72 (m, 15H), 7.18–7.15 (m, 1H), 5.44 (q, *J* = 15.3, 12.0 Hz, 1H), 5.10 (d, *J* = 10.6 Hz, 1H), 4.62 (t, *J* = 7.3 Hz, 2H), 4.20–4.14 (m, 1H), 4.08–3.97 (m, 6H), 3.62–3.52 (m, 4H), 3.37 (d, *J* = 7.9 Hz, 2H), 3.16 (s, 4H), 3.10 (s, 6H), 2.95–2.88 (m, 1H), 2.69–2.62 (m, 1H), 2.46–2.11 (m, 8H), 2.11–1.92 (m, 7H), 1.50 (s, 3H), 1.47–1.32 (m, 6H), 1.30 (s, 3H), 1.24 (s, 3H), 1.15–0.95 (m, 17H), 0.89 (s, 3H), 0.85 (s, 3H), 0.80 (d, *J* = 6.7 Hz, 3H). ^13^C-NMR (101 MHz, DMSO-*d_6_*) δ 171.12, 169.83, 169.46, 168.61, 163.83, 163.15, 156.51, 148.71, 135.13, 135.10, 133.60, 133.49, 132.17, 131.82, 131.51, 130.42, 130.29, 129.73, 128.76, 128.46, 124.98, 123.47, 122.99, 118.40, 117.82, 113.56, 112.99, 73.58, 69.19, 65.74, 58.08, 57.89, 54.62, 51.14, 48.94, 48.58, 48.16, 44.36, 43.54, 42.08, 38.59, 36.66, 36.42, 36.33, 36.26, 36.16, 35.84, 31.78, 30.21, 27.01, 25.55, 23.46, 23.33, 21.07, 20.80, 20.67, 20.08, 19.64, 18.37, 17.84, 17.71, 17.49, 16.25. HRMS (ESI) *m/z*: 1352.7621 [M]^+^, calcd. for [C_81_H_105_N_6_O_10_P]^+^ 1352.7624.

### 4.2. EPR Spectroscopy and Sample Preparation

X-Band (~9.43 GHz) room temperature CW-EPR measurements were performed on a Magnettech MiniScope MS400 benchtop spectrometer (Magnettech, Berlin, Germany). Spectra were recorded with a microwave power of 3.16 mW, 100 kHz modulation frequency, modulation amplitude of 0.1mT and 4096 points. The final spectra were accumulations of 10 scans, each took 60 s. The samples were dissolved in methanol. Therefore, to reduce the line broadening effect due to the dissolved oxygen in the solvent, all samples were flushed with argon before EPR measurements.

### 4.3. Biology

#### 4.3.1. Cell Lines and Cultivation

PC3 and HT29 cell lines were supplied by the Leibniz Institute of Plant Biochemistry. The cells were grown in RPMI 1640 completed medium (supplemented with 10% FCS, 1% glutamine, and 1% penicillin/streptomycin) at 37 °C and 5% CO_2_. Cells were seeded at 5 × 103 cells/well in 96-well plates for viability determination and 1.5 × 105 cells/well in 6-well plates for flow cytometry and western blotting.

#### 4.3.2. MTT and CV Assays

For the fast screening the two cell lines were treated with 0.1 and 10 µM of the synthesized compounds 6-12, and 18 for 48 h. The compounds which showed anticancer activity was further analyzed to determine their IC_50_, in which, each compound was tested in 7 different concentrations (100, 50, 25, 12.5, 6.25, 3.125, 1.56 µM) for 48 h. Afterward, for the CV assay, the cells were fixed by 4% paraformaldehyde for 15 min at RT and then the cells were stained with 0.1% CV solution for 15 min. Subsequently, the cells were washed with dd H_2_O, dried overnight and the dye was dissolved using 33% acetic acid. For MTT assay, the cells were incubated with MTT (0.5 mg/mL) for 20 min. Then, the MTT solution was removed and the dye was dissolved using DMSO. The dissolved dyes were measured using an automated microplate reader (Spectramax, Molecular Devices, San José, CA, USA) at 570 nm with a background wavelength of 670 nm. The IC_50_ values were calculated using the four-parameter logistic function and presented as the mean and all assays were performed in three biological replicates. The cell viability was expressed as a percentage compared to a negative control which was cells treated with complete medium and a positive control which was cells treated with digitonin (125 µM) [41,42].

#### 4.3.3. Apoptosis Analysis

The PC3 cells were prepared in a 6 well plate, treated with IC_50_ and 2 × IC_50_ of compound **8** (7.4 and 14.9 µM), and incubated for 48 h at 37 °C and 5% CO_2_. After the incubation, cells were stained by AnnV and PI (5 µL of AnnV, 2 µL of PI in 100 µL PBS) to determine apoptosis using flow cytometry (FACSAria III, BD Biosciences, Franklin Lakes, NJ, USA). The procedure was carried out according to the manufacturer’s supplied instructions [42].

#### 4.3.4. Cell Cycle Analysis

The PC3 cells were prepared in a 6 well-plate and treated with IC_50_ and 2 × IC_50_ of compound **8** (7.4 and 14.9 µM) and incubated for 48 h at 37 °C and 5% CO_2_. Afterward, the cells were fixed in 70% ethanol overnight at 2 °C and then, stained with 1 µg/mL of DAPI at room temperature for 10 min. At last, the cells were analyzed by flow cytometry (FACSAria III) [42].

#### 4.3.5. Western Blot Analysis

PC3 cells were cultivated with an IC_50_ dose of **8** for 2 h, 6 h, 12 h, 24 h, and 48 h. The cell lysis was performed using protein lysis buffer (62.5 mMTris–HCl (pH 6.8), 2% (*w*/*v*) SDS, 10% glycerol, and 50 mM dithiothreitol). The proteins were electrically separated using 12% SDS-polyacrylamide gels where a PageRuler prestained ladder was used as a protein molecular weight marker. The proteins were electrically transferred to nitrocellulose membranes by western blot system (Owl HEP-1, ThermoFisher Scientific, Schwerte, Germany). The membranes were blocked by 5% (*w*/*v*) BSA in PBS with 0.1% Tween 20 for 1 h at RT. Afterwards, blots were incubated overnight at 4 °C with α/β-Tubulin rabbit Ab, Caspase-3 rabbit Ab, β-actin rabbit Ab, and Bcl-XL rabbit Ab. As a secondary antibody Anti-rabbit IgG, HRP-linked Antibody was used. Bands were visualized using an ECL Prime Western Blotting System.

#### 4.3.6. Investigation of ROS Production

For the detection of reactive oxygen and nitrogen species, PC3 cells were stained with 1 µM of DHR solution in 0.1% PBS for 10 min. Afterwards, the cells were treated with IC_50_ and 2 × IC_50_ of compound **8** for 48 h. After 48 h, cells were trypsinized, washed with PBS, and then analyzed with flow cytometry [43,44].

### 4.4. Microscopy

#### Fluorescent Microscopy

PC3 cells were seeded in a 6-well plate for 24 h at 37 °C and 5% CO_2_. Afterward, cells were stained with 0.1 µM of MitoTracker^TM^ Deep Red in a complete medium for 15 min (based on the manufacturer’s protocol). The cells were washed twice with PBS. After washing, cells were treated with the IC_50_ of the tested compound for 24 h. The cells were washed twice with PBS, upon which 1 mL of medium was added. Finally, the cells were observed using GFP and Texas Red channels using LSM700 (Carl Zeiss, Jena, Germany) and EVOS FL AUTO (ThermoFisher, Schwerte, Germany).

## Figures and Tables

**Table 1:** IC_50_ values (µM) for the most active compounds against HT29 and PC3 cell lines determined by MTT and CV assays.

Compound	PC3		HT29	
	CV	MTT	CV	MTT
**1**	24.64 ± 1.78	25.43 ± 4.35	24.97 ± 0.57	19.02 ± 2.26
**6**	13.69 ± 0.80	7.43 ± 0.72	13.16 ± 0.97	8.98 ± 0.43
**7**	10.59 ± 0.85	10.54 ± 0.91	13.82 ± 0.29	11.87 ± 0.94
**8**	7.44 ± 0.80	6.00 ± 1.09	8.10 ± 0.43	7.41 ± 0.56
**9**	15.26 ± 1.01	13.85 ± 2.04	6.98 ± 0.25	12.94 ± 1.03
**18**	9.27 ± 0.73	6.19 ± 0.20	16.30 ± 0.87	12.23 ± 0.67

## Supplementary Materials

The following are available online at https://www.mdpi.com/article/10.3390/ijms22137125/s1. Table S1. EPR charactersitics for nitroxides 6-12, 14, 16-18; ^1^H-, ^13^C-NMR spectra and HPLC-profiles of products 6-12, 14, 16-18.
