# Supplementary material for: Access to New Cytotoxic Triterpene and Steroidal Acid-TEMPO Conjugates by Ugi Multicomponent-Reactions
Source: Int J Mol Sci. 2021 Jul 1;22(13):7125. doi: 10.3390/ijms22137125 (PMC8268079; doi:10.3390/ijms22137125)
Supplement: Supplementary file 1 [file ijms-22-07125-s001.zip › ijms-1237369-supplementary.pdf]

Supplementary Materials for

Access to new cytotoxic triterpene and steroidal acid-TEMPO conjugates by Ugi multicomponent-reactions

Table S1. Numerical values of EPR charactersitics (A_{iso} and τc (ns)) for the nitroxide conjugated compounds, obtained by simulations.

Compound	τc(ns)	A_{iso}(MHz)
6	1,10	45,33
7	1,39	45,33
8	2,00	47,33
9	2,52	47,33
10	2,00	47,67
11	2,28	47,67
12	2,44	45,33
14	1,30	45,33
16	1,93	45,33
17	2,07	45,33
18	2,68	45,33

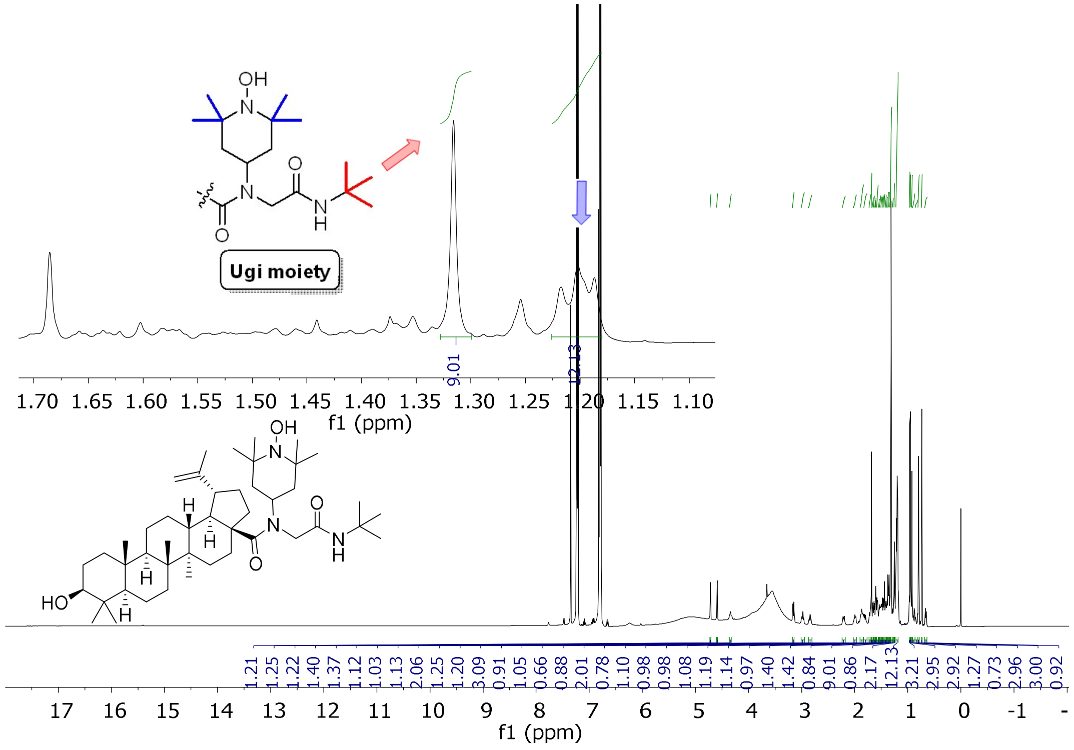

Figure S2. ^{1}H NMR (600 MHz, $CDCl_3$) spectrum of compound **6**.

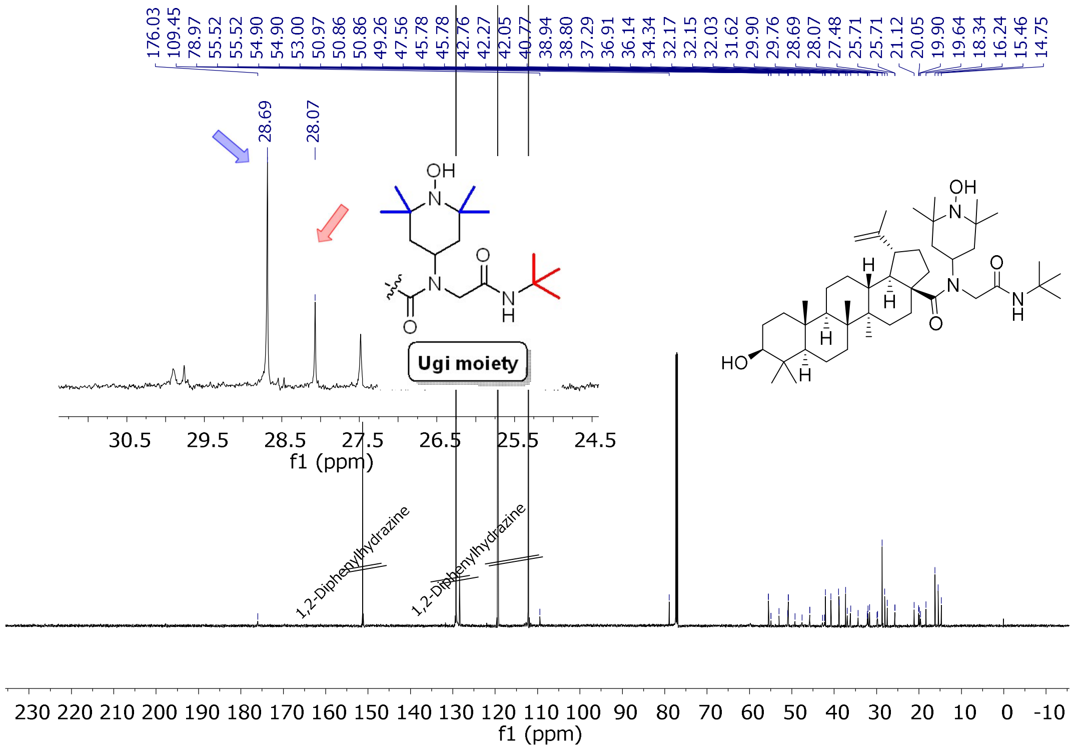

Figure S3. ^{13}C NMR (151 MHz, $CDCl_3$) spectrum of compound **6**.

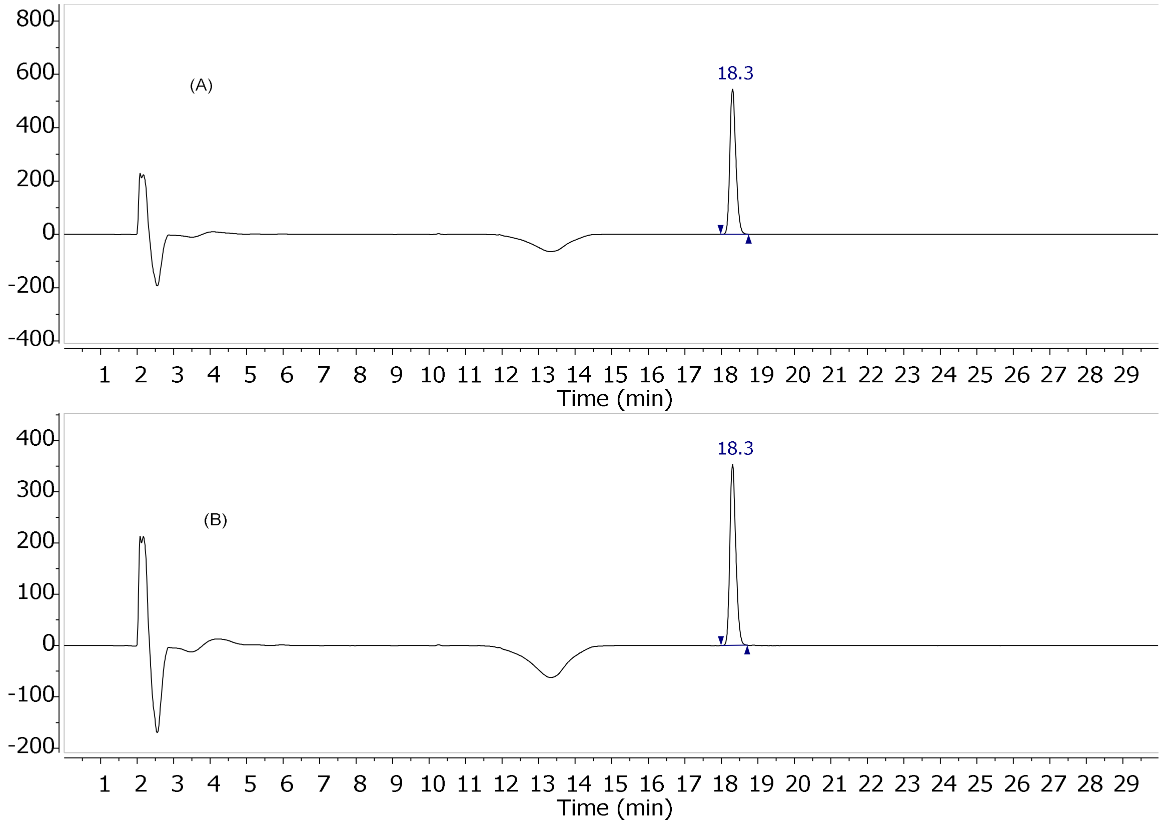

Figure S4. RP-HPLC chromatogram of compound **6** a) 210 nm, b) 215 nm.

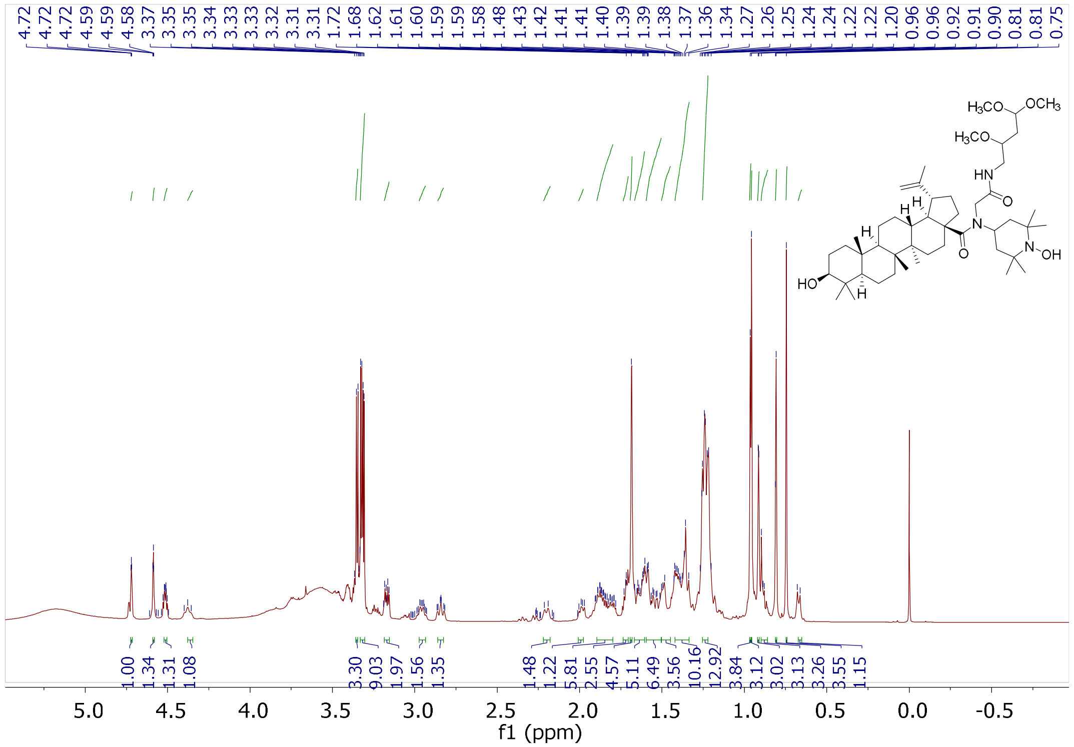

Figure S5. ^{1}H NMR (600 MHz, $CDCl_3$) spectrum of compound **7**.

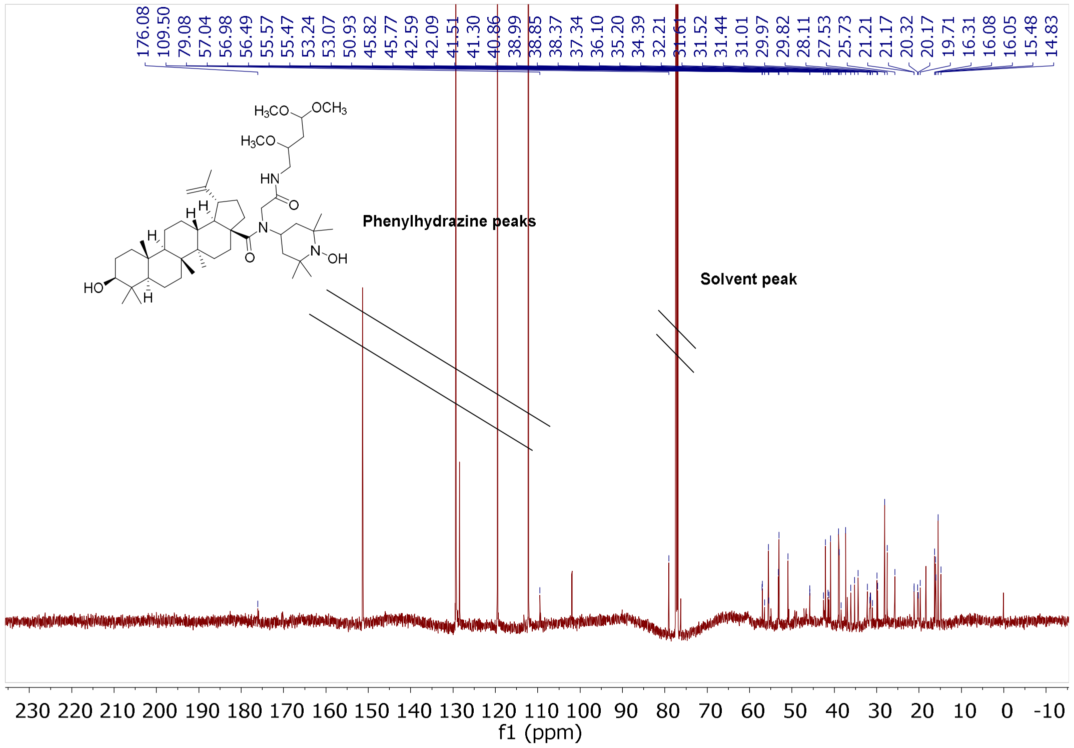

Figure S6. ^{13}C NMR (151 MHz, $CDCl_3$) spectrum of compound **7**.

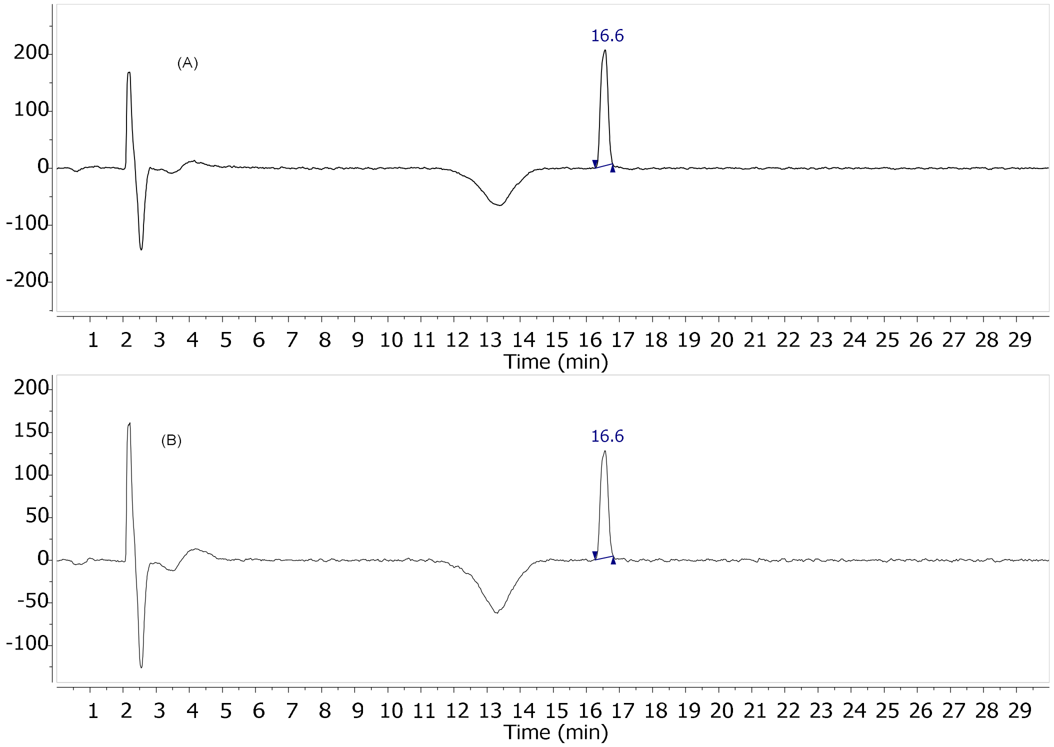

Figure S7. RP-HPLC chromatogram of compound **7** a) 210 nm, b) 215 nm.

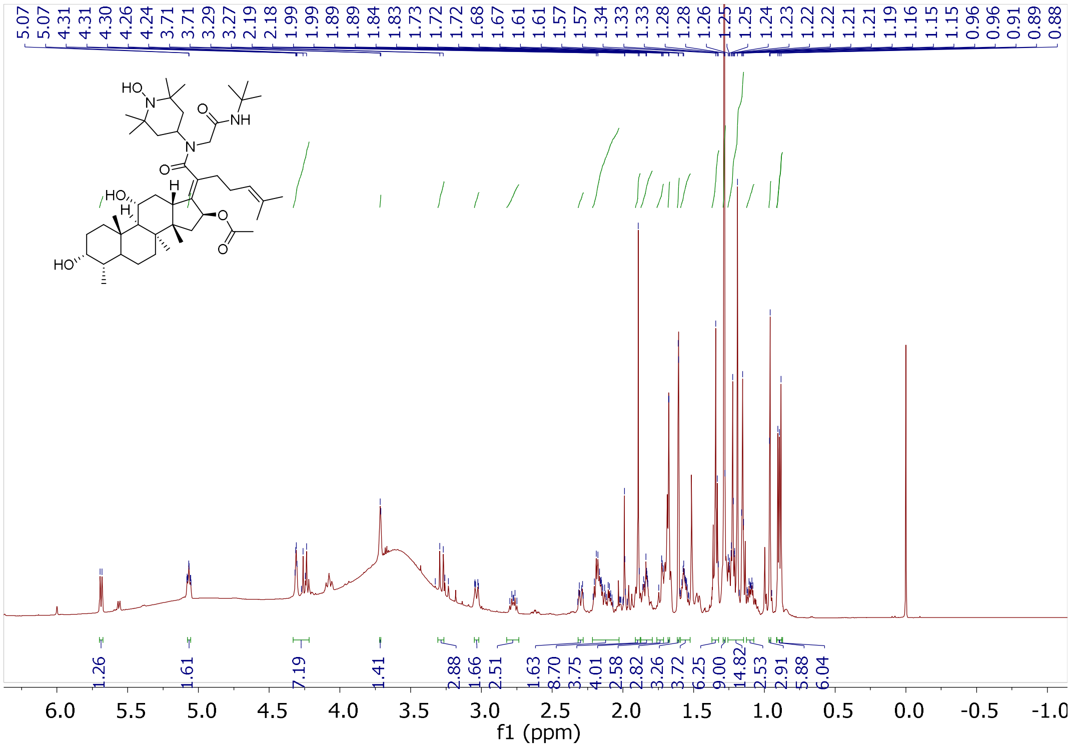

Figure S8. ^{1}H NMR (600 MHz, $CDCl_3$) spectrum of compound **8**.

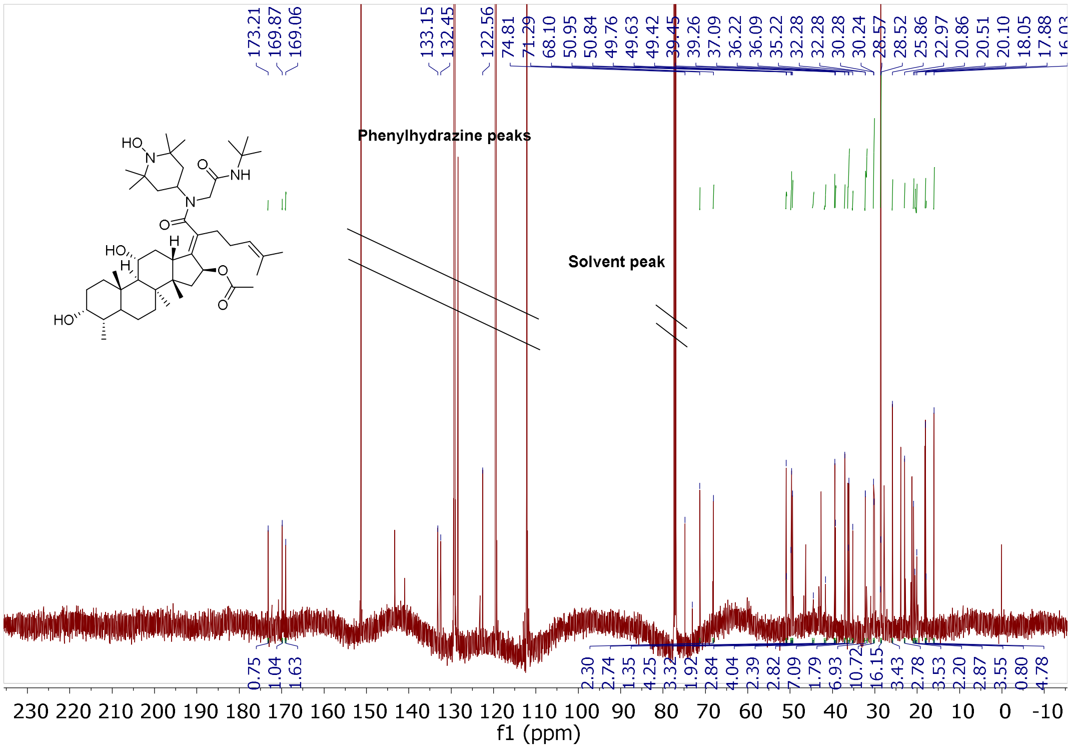

Figure S9. ^{13}C NMR (151 MHz, $CDCl_3$) spectrum of compound **8**.

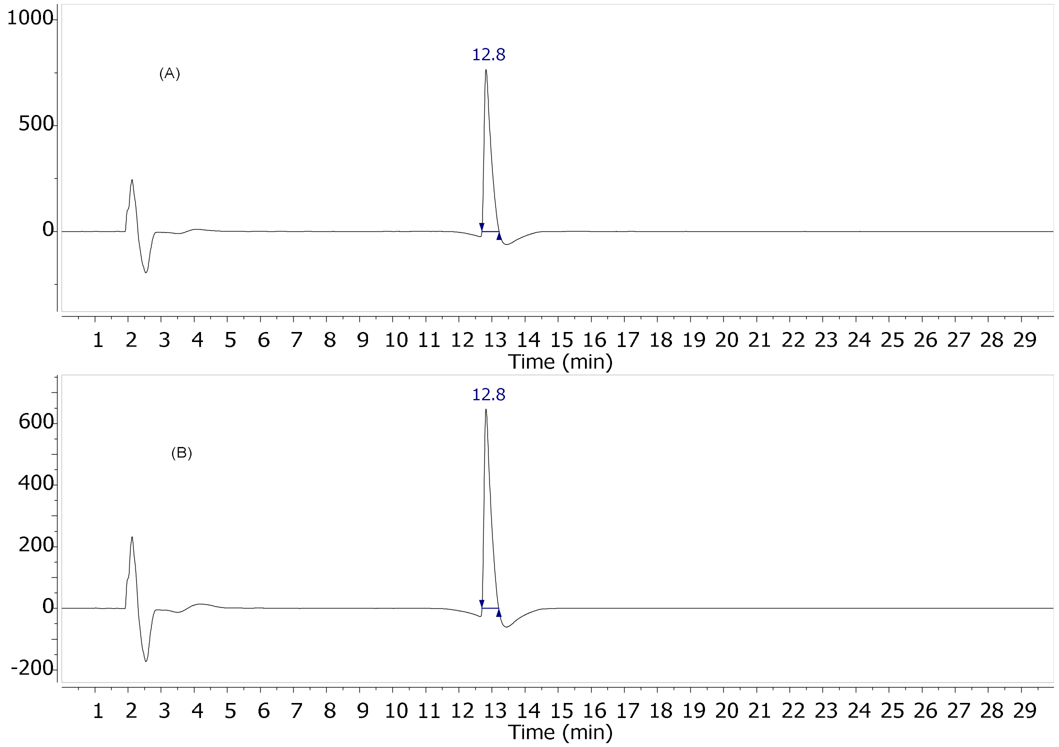

Figure S10. RP-HPLC chromatogram of compound **8** a) 210 nm, b) 215 nm.

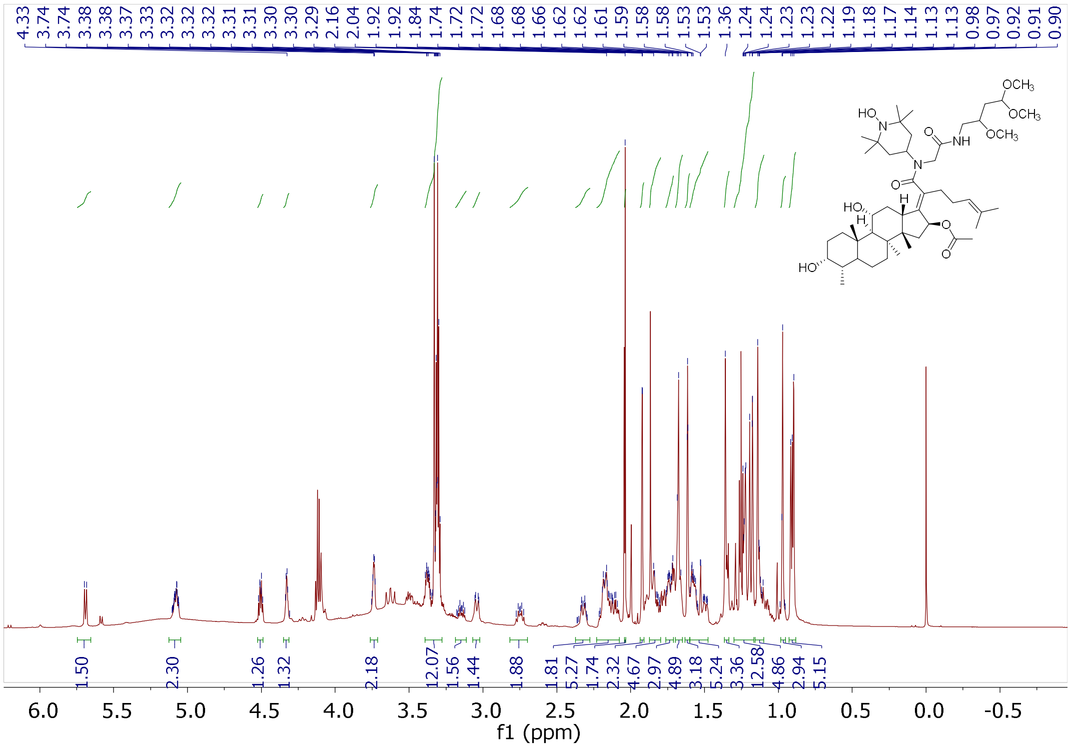

Figure S11. ^{1}H NMR (600 MHz, $CDCl_3$) spectrum of compound **9**.

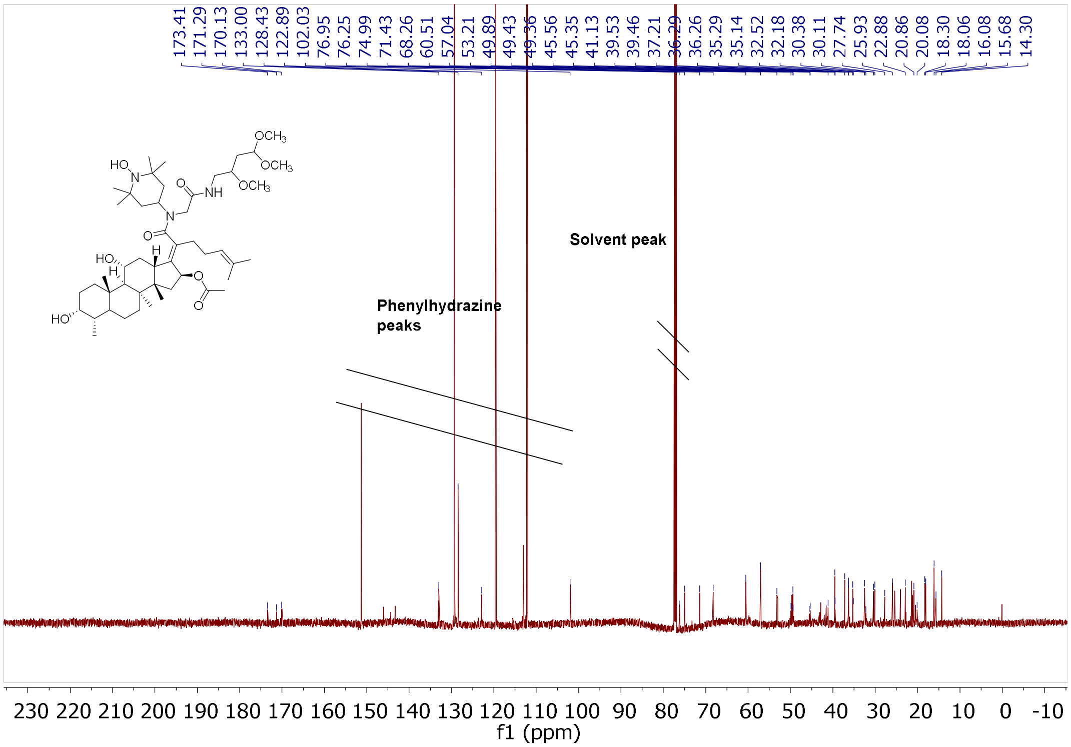

Figure S12. ^{13}C NMR (151 MHz, $CDCl_3$) spectrum of compound **9**.

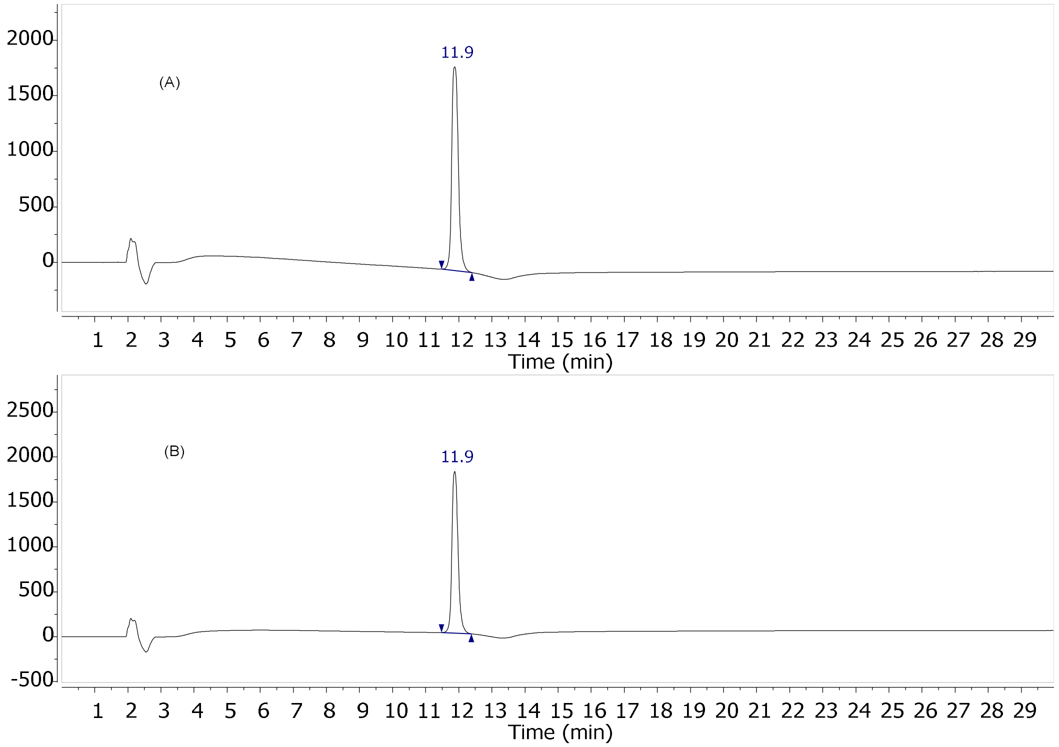

Figure S13. RP-HPLC chromatogram of compound **9** a) 210 nm, b) 215 nm.

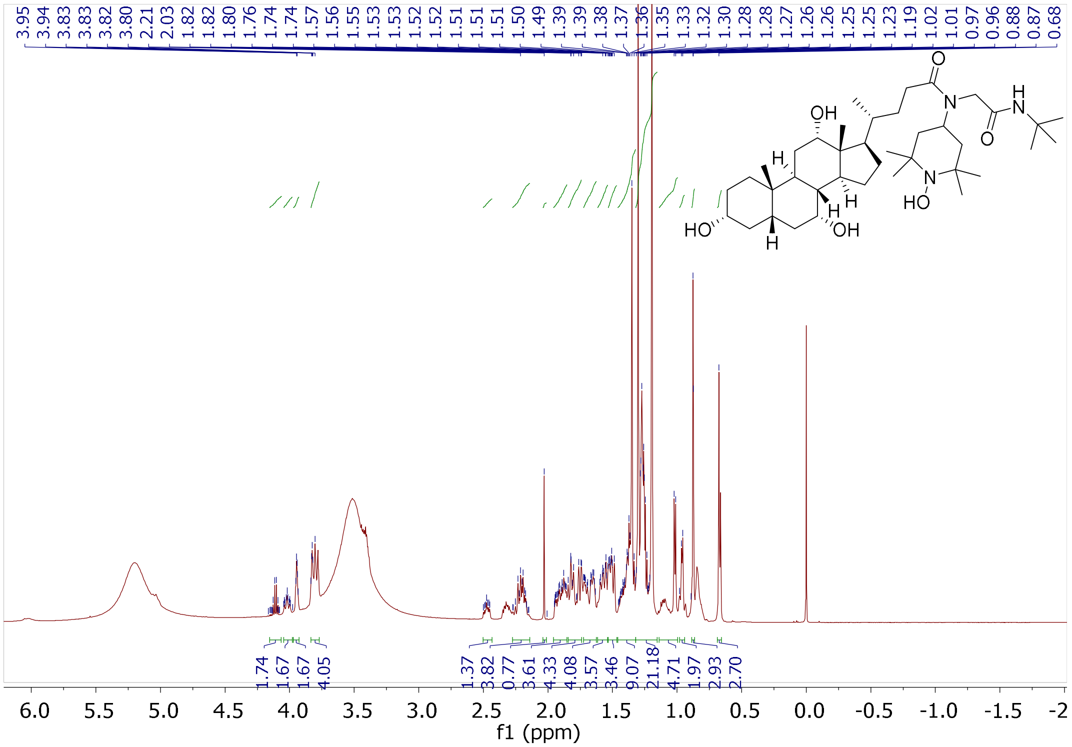

Figure S14. ^{1}H NMR (600 MHz, $CDCl_3$) spectrum of compound **10**.

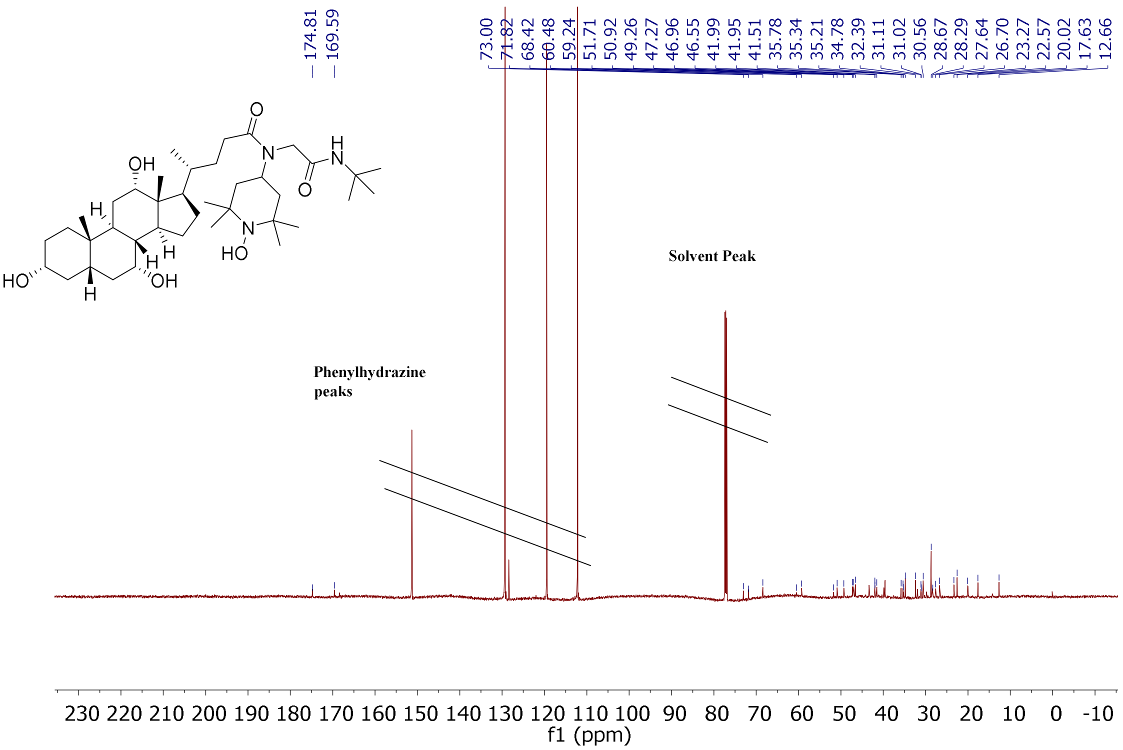

Figure S15. ^{13}C NMR (151 MHz, $CDCl_3$) spectrum of compound **10**.

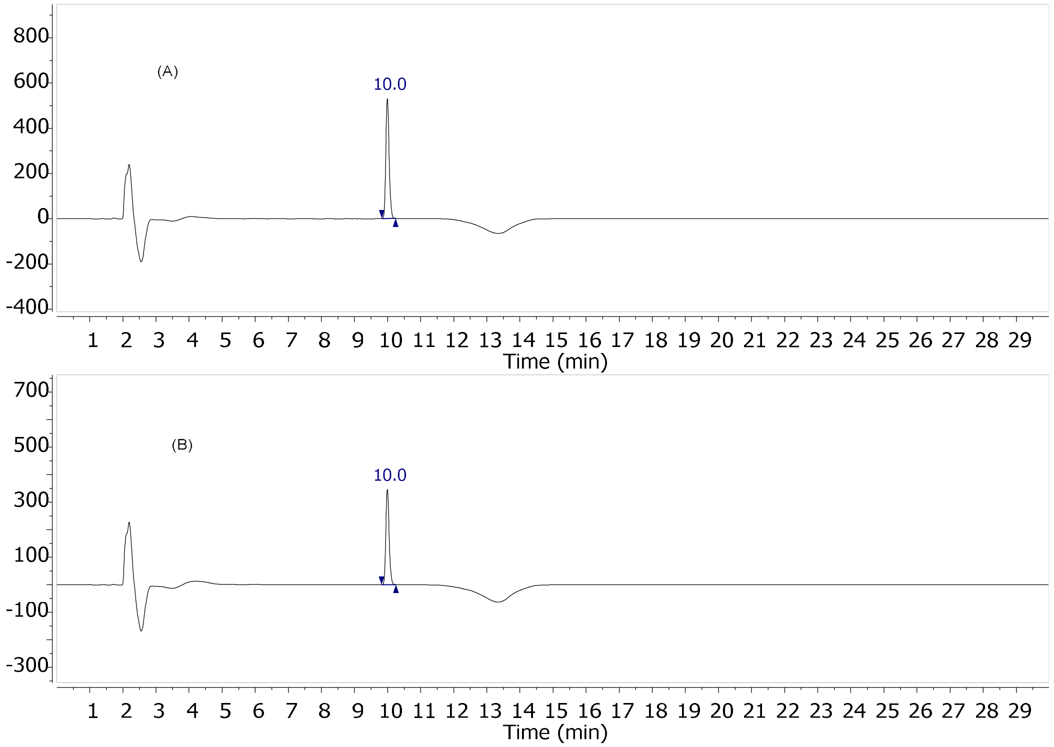

Figure S16. RP-HPLC chromatogram of compound **10** a) 210 nm, b) 215 nm.

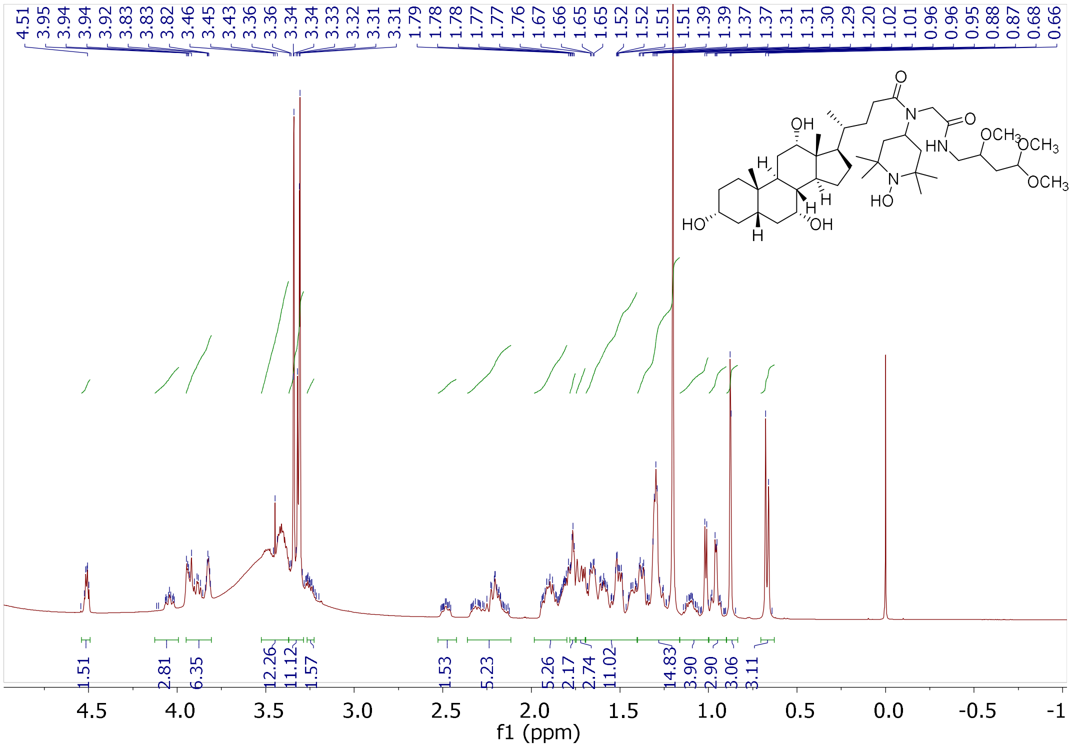

Figure S17. ^{13}C NMR (151 MHz, $CDCl_3$) spectrum of compound **11**.

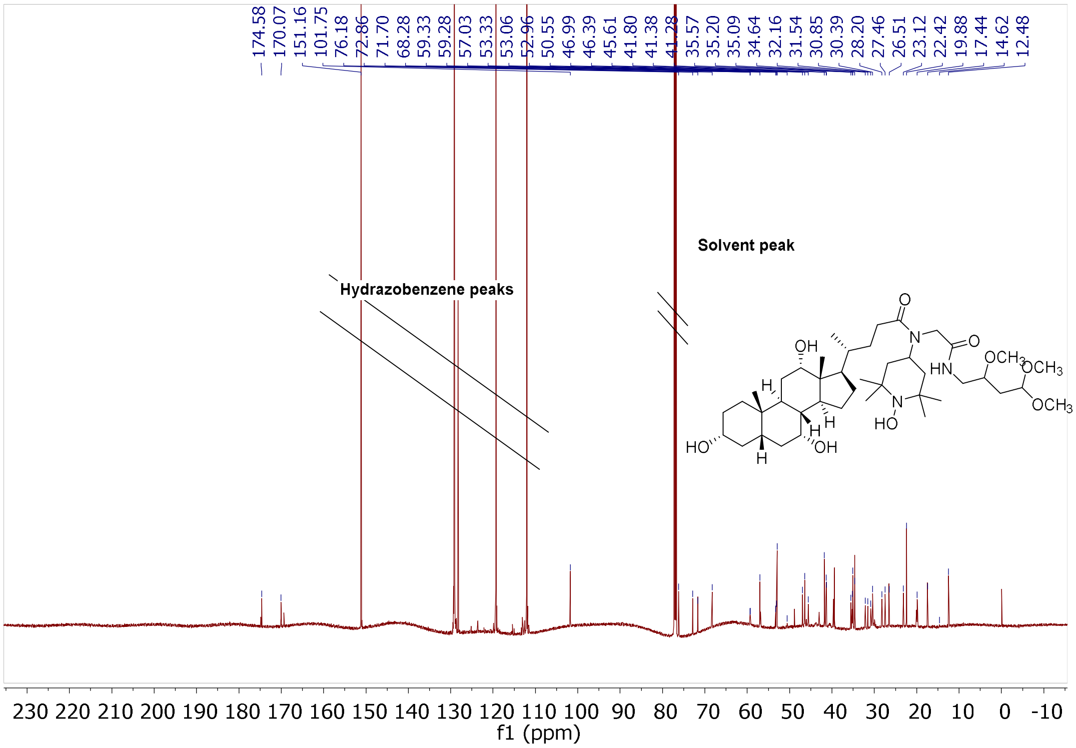

Figure S18. ^{13}C NMR (151 MHz, $CDCl_3$) spectrum of compound **11**.

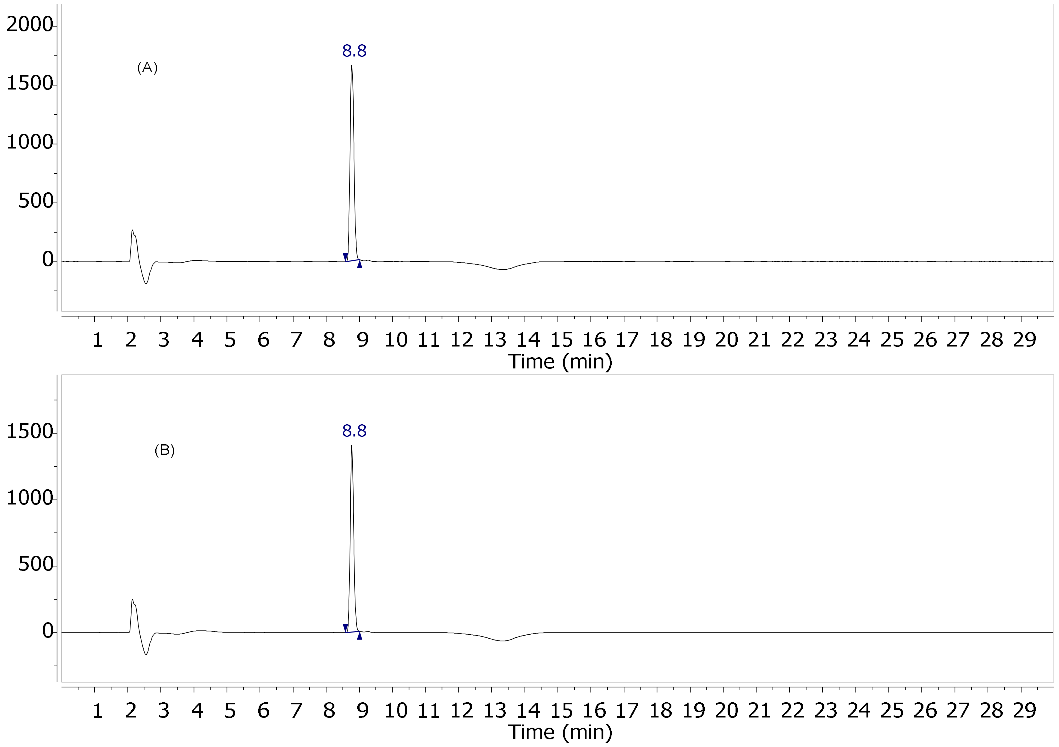

Figure S19. RP-HPLC chromatogram of compound **11** a) 210 nm, b) 215 nm.

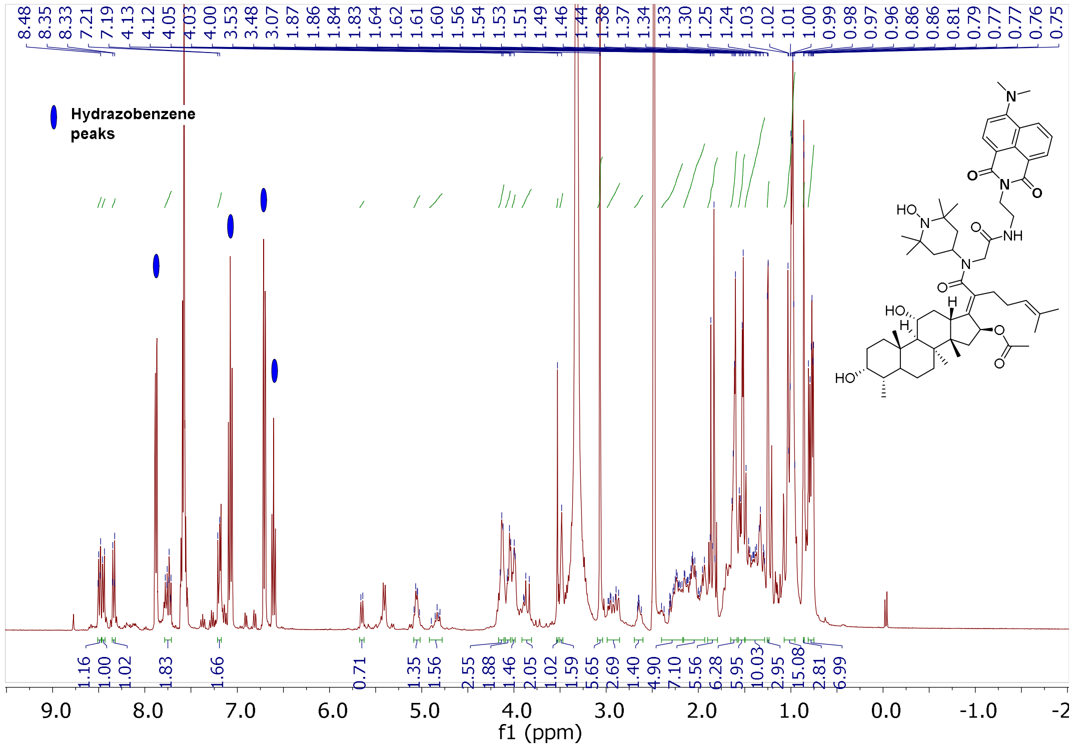

Figure S20. ^{1}H NMR (400 MHz, DMSO-d_6) spectrum of compound **12**.

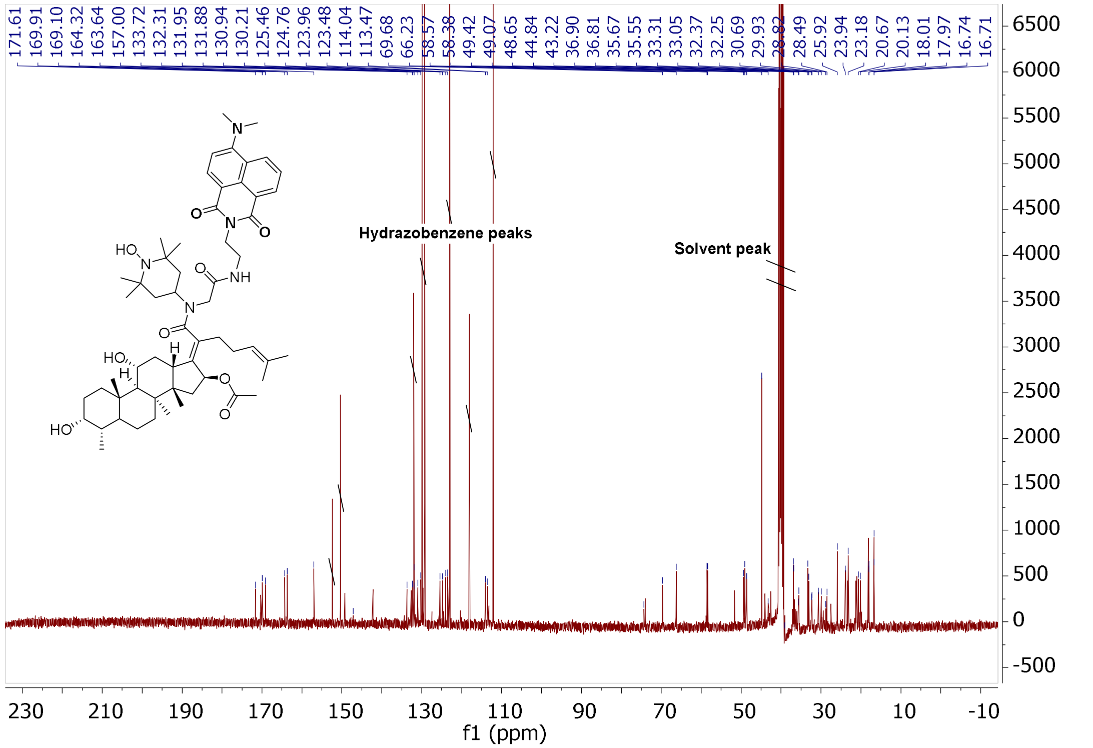

Figure S21. ^{13}C NMR (101 MHz, DMSO-d_6) spectrum of compound **12**.

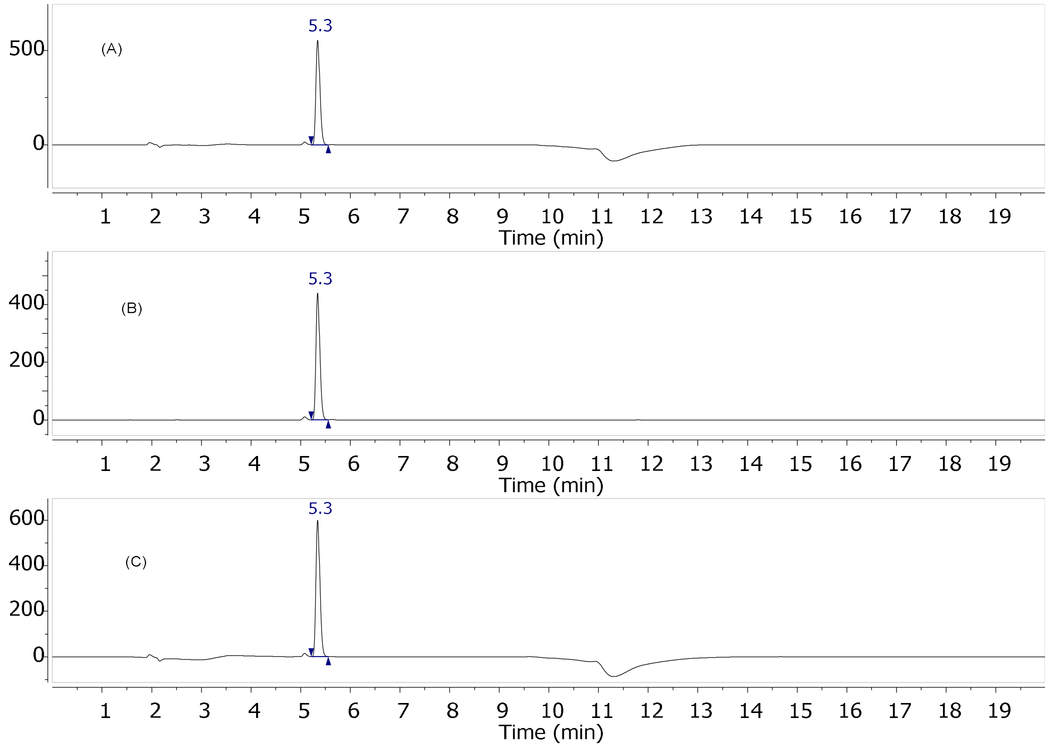

Figure S22. RP-HPLC chromatogram of compound **12** a) 210 nm, b) 215 nm, c) 254 nm.

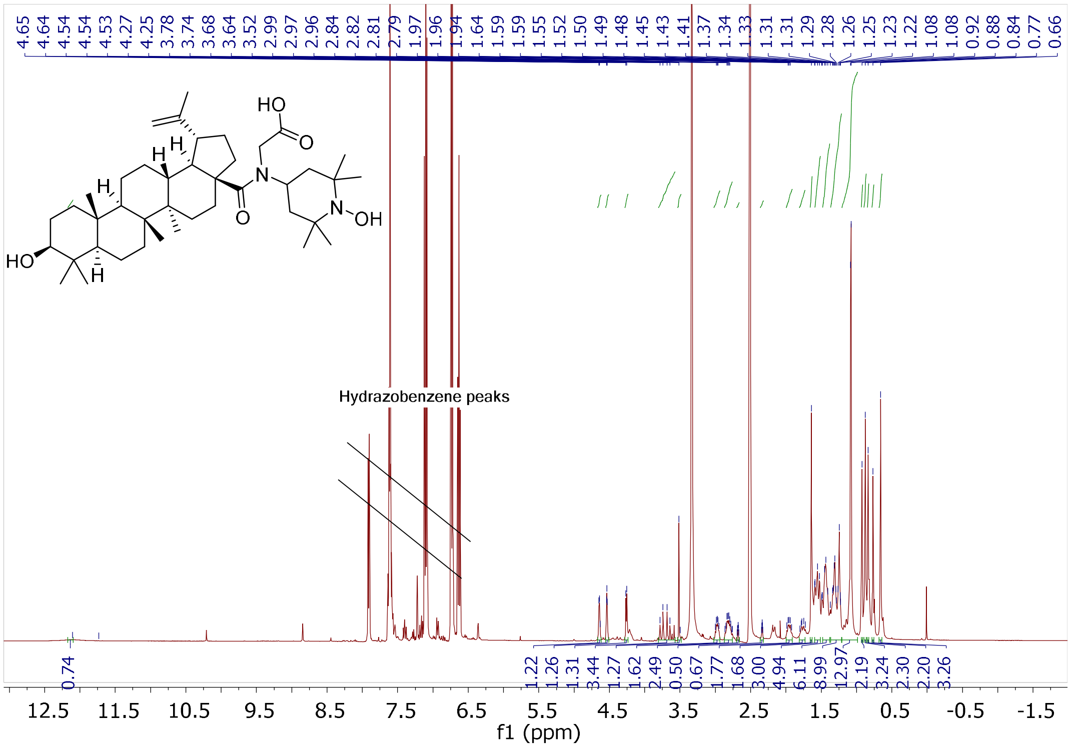

Figure S23. ^{1}H NMR (400 MHz, DMSO-*d6*) spectrum of compound **14**.

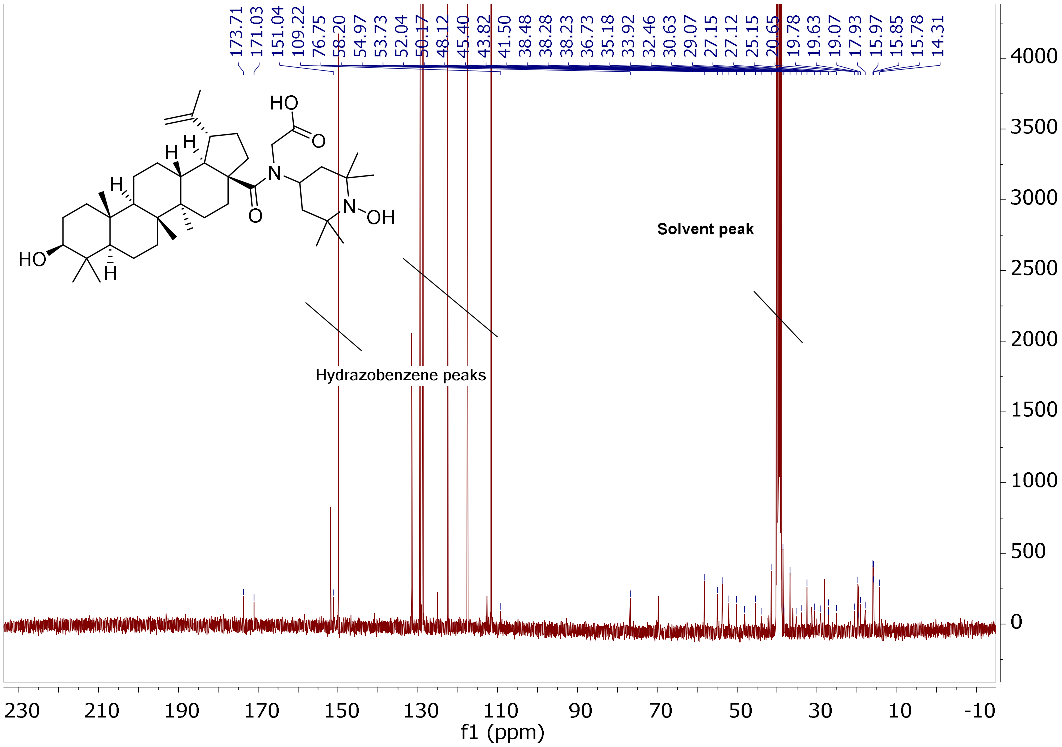

Figure S24. ^{13}C NMR (101 MHz, DMSO-d_6) spectrum of compound **14**.

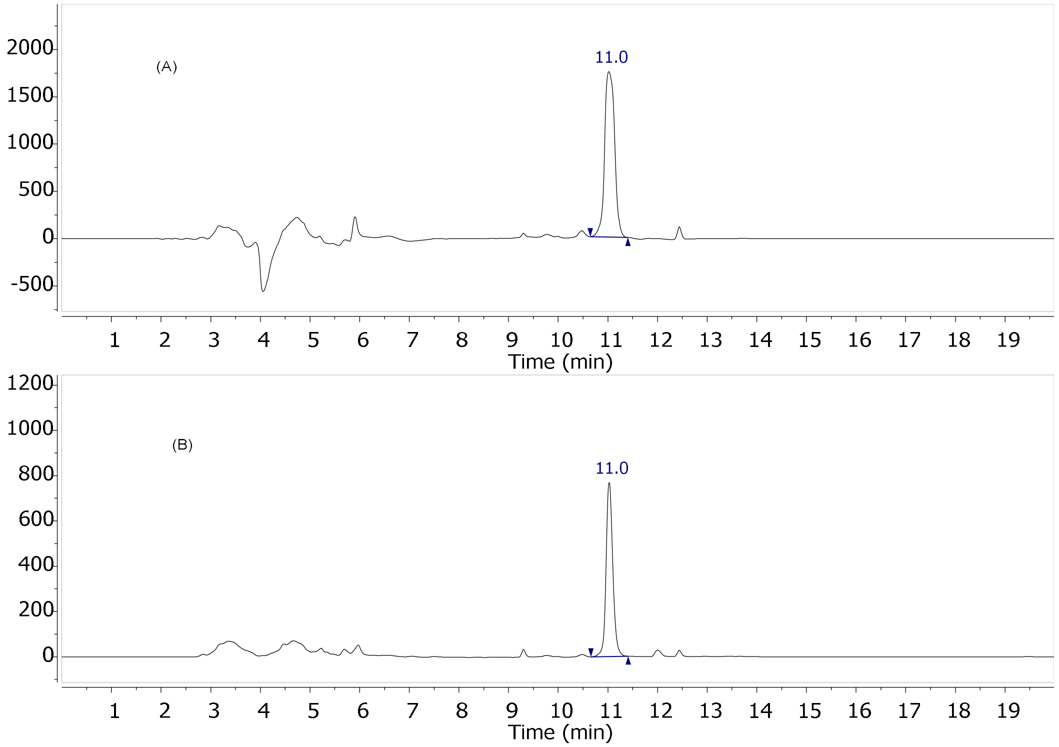

Figure S25. RP-HPLC chromatogram of compound **14** a) 210 nm, b) 215 nm.

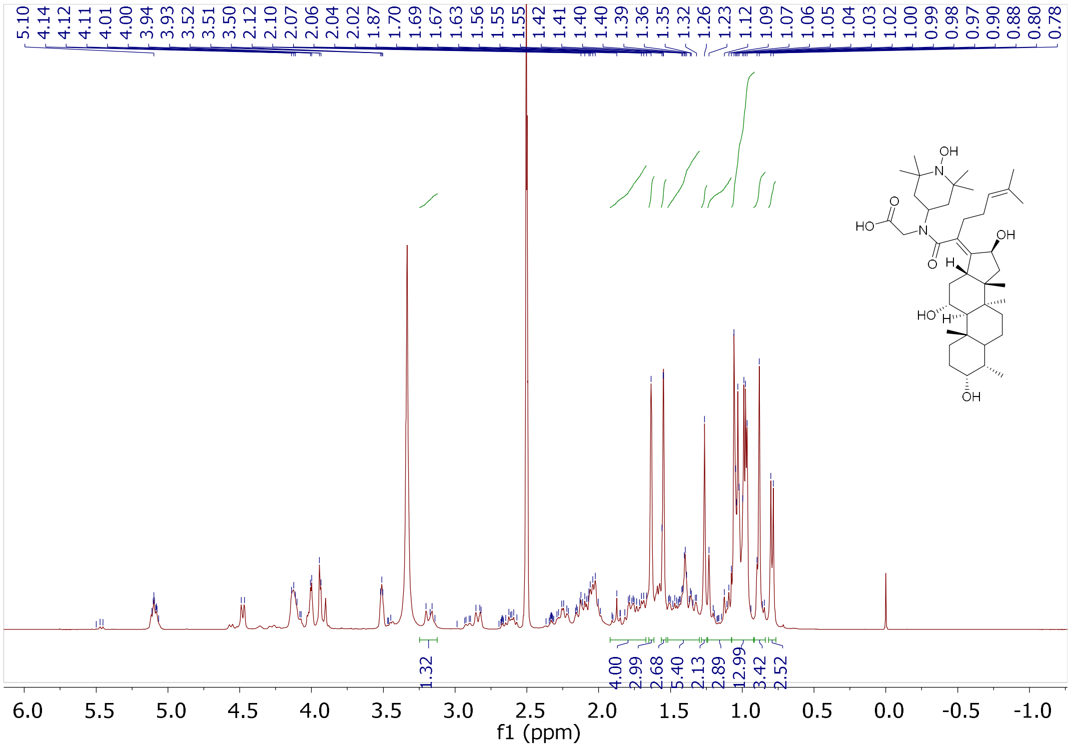

Figure S26. ^{1}H NMR (400 MHz, DMSO-*d6*) spectrum of compound **16**.

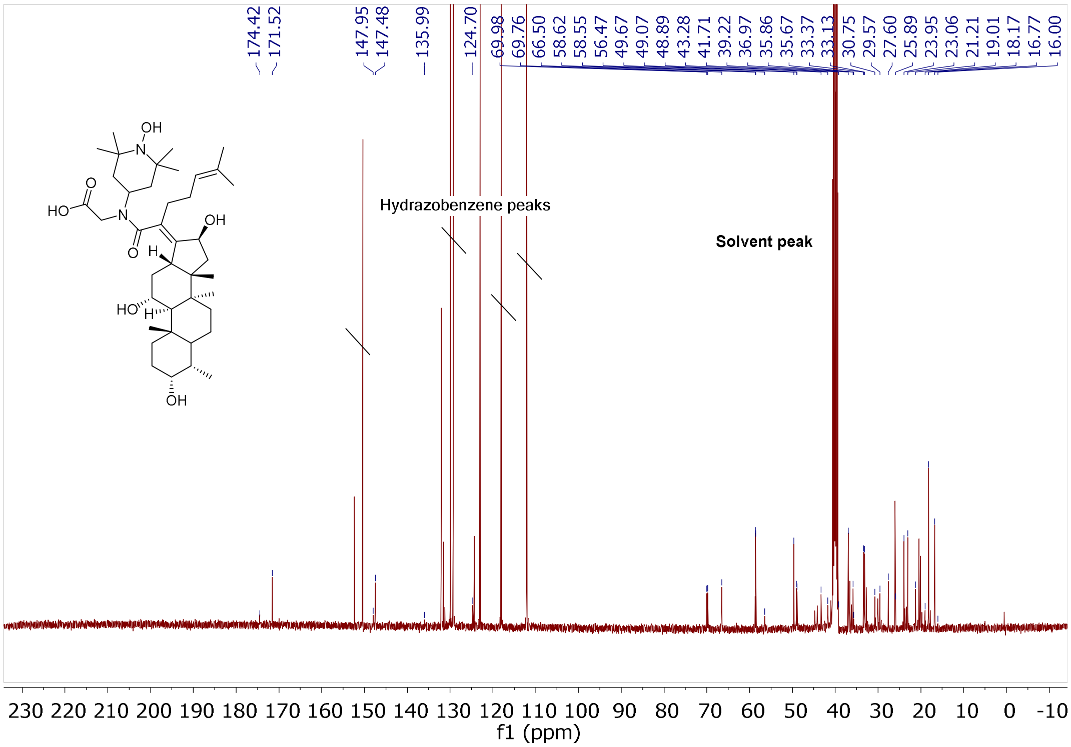

Figure S27. ^{13}C NMR (101 MHz, DMSO-d_6) spectrum of compound **16**.

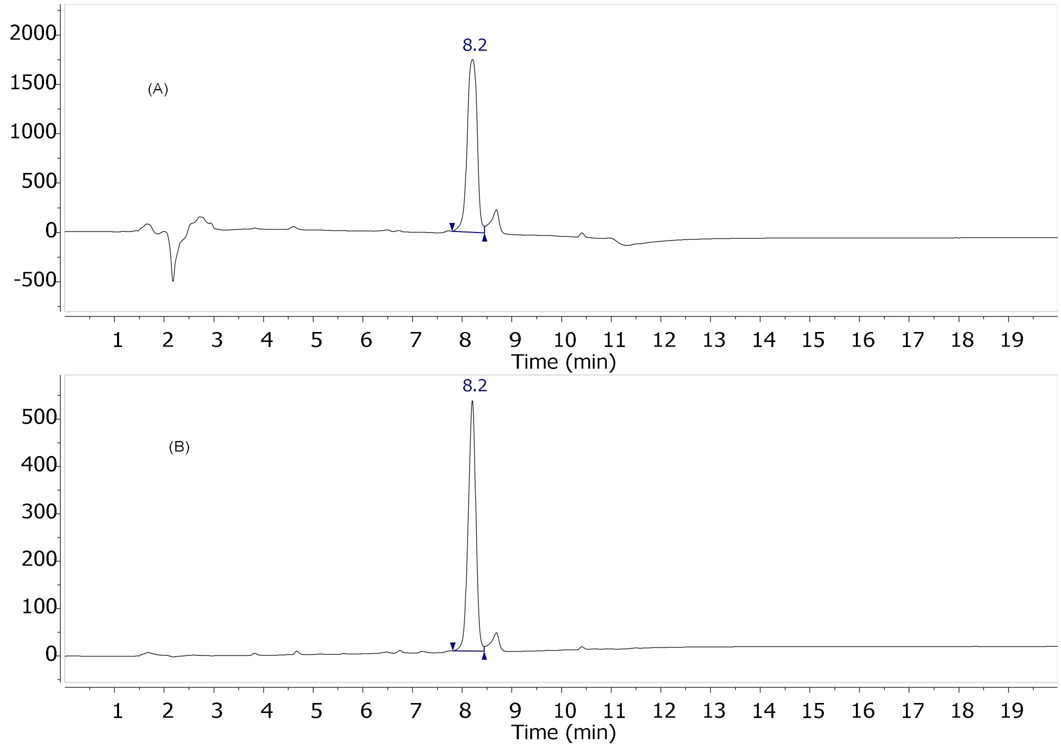

Figure S28. RP-HPLC chromatogram of compound **16** a) 210 nm, b) 215 nm.

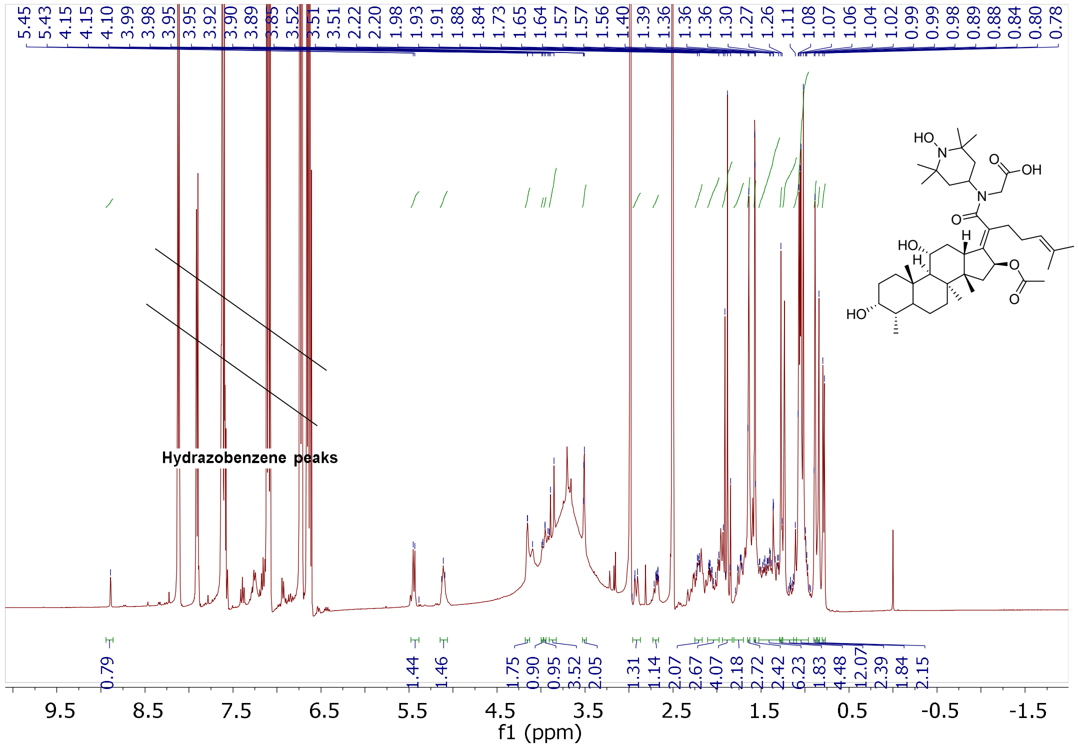

Figure S29. ^{1}H NMR (400 MHz, DMSO-*d6*) spectrum of compound **17**.

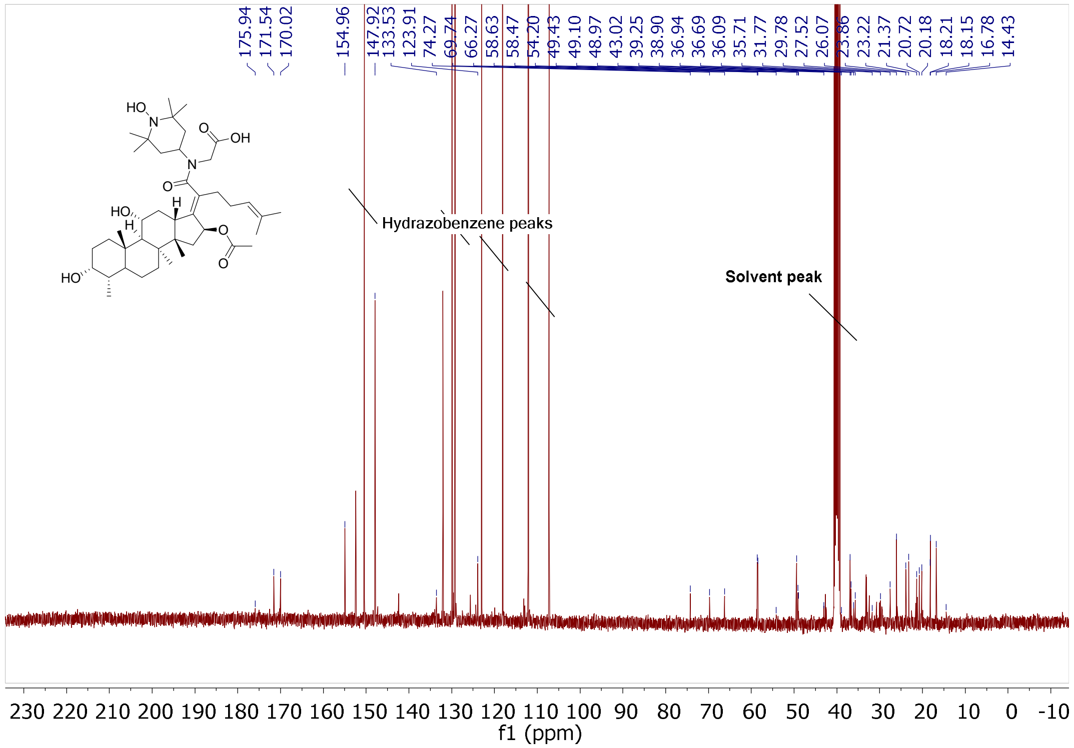

Figure S30. ^{13}C NMR (101 MHz, DMSO-d_6) spectrum of compound **17**.

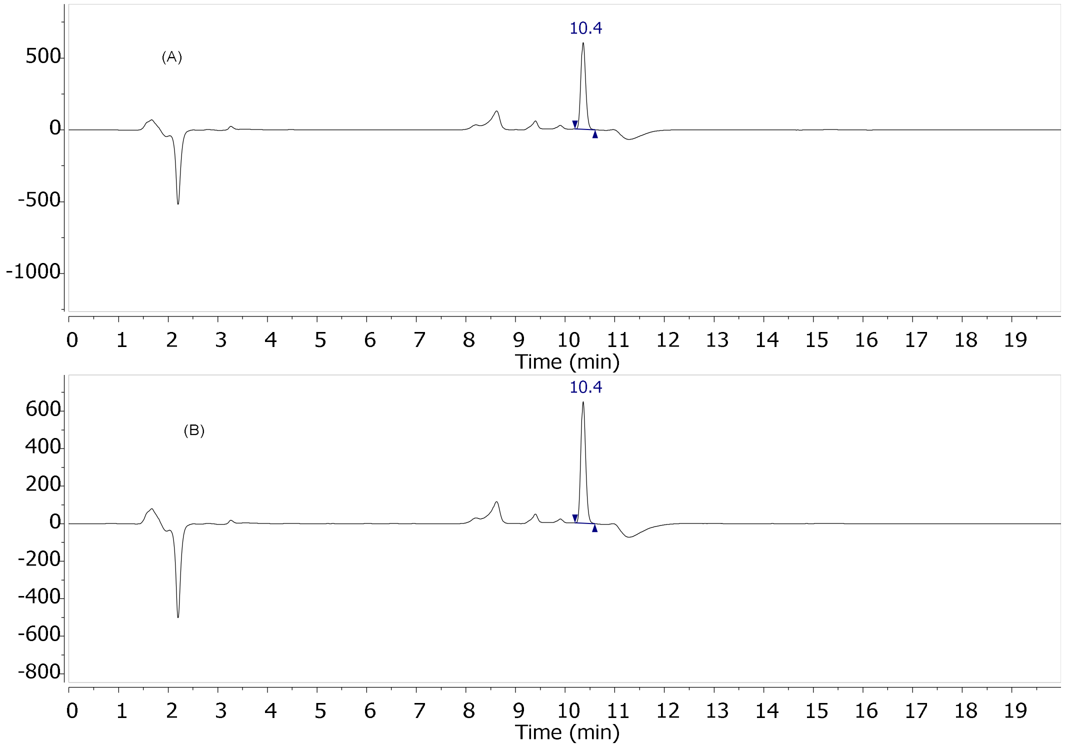

Figure S31. RP-HPLC chromatogram of compound **17** a) 210 nm, b) 215 nm.

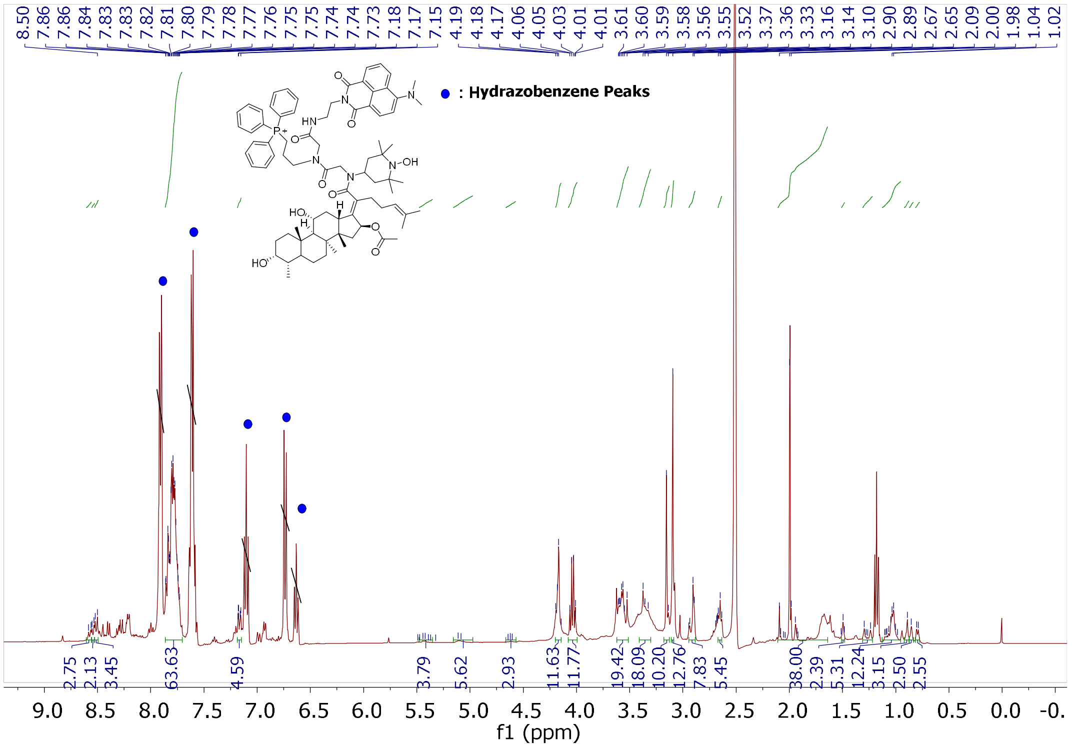

Figure S32. ^{1}H NMR (400 MHz, DMSO-*d6*) spectrum of compound **18**.

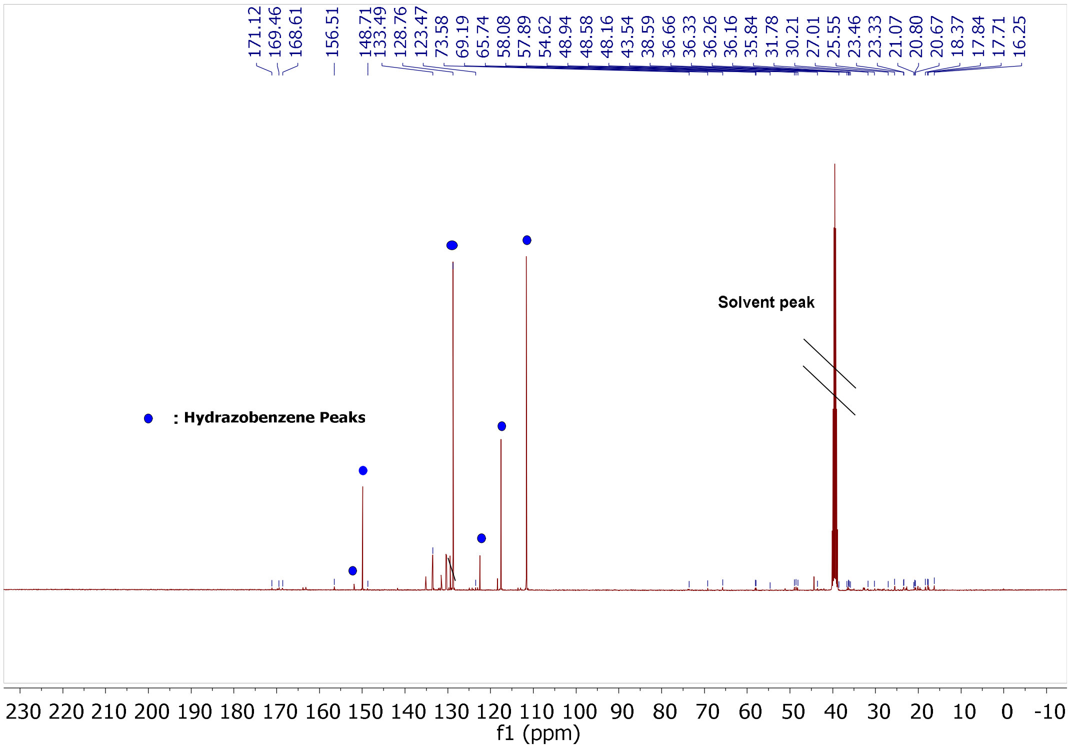

Figure S33. ^{13}C NMR (101 MHz, DMSO-d_6) spectrum of compound **18**.

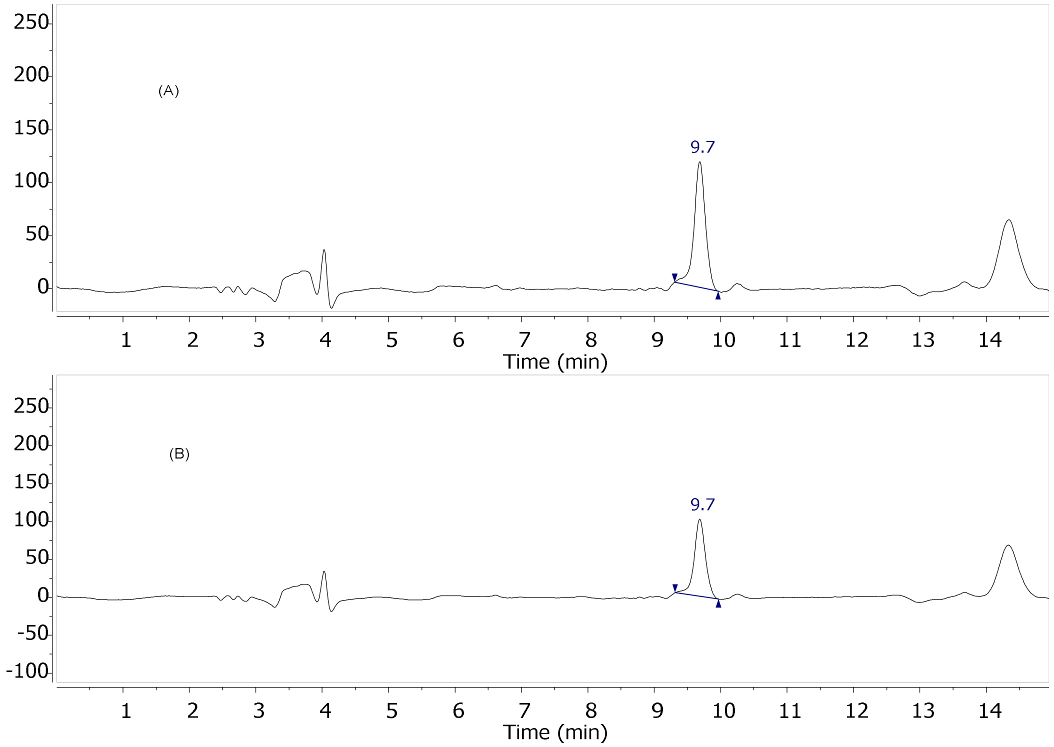

Figure S34. RP-HPLC chromatogram of compound **18** a) 210 nm, b) 215 nm.
